# The cytokinin histidine kinase receptors regulate nodulation, shoot and root development in *Pisum sativum*

**DOI:** 10.3389/fpls.2026.1750990

**Published:** 2026-03-16

**Authors:** Karen Velandia, Alejandro Correa-Lozano, Alex Tomkinson, Stephane Boivin, Tracy François, Marion Dalmais, Anthony Klein, Christine Le Signor, Abdelhafid Bendahmane, Florian Frugier, James B. Reid, Eloise Foo

**Affiliations:** 1 Discipline of Biological Sciences, School of Natural Sciences, University of Tasmania, Hobart, TAS, Australia; 2 Institute of Plant Sciences Paris Saclay (IPS2), Centre National de la Recherche Scientifique (CNRS), Paris-Saclay University, Paris-Cité University, Institut National de Recherche pour l’Agriculture, l’alimentation et l’Environnement (INRAE), Univ d’Evry, Gif-sur-Yvette, France; 3 Université Bourgogne Europe, Institut Agro Dijon, INRAE, Agroécologie, Dijon, France

**Keywords:** cytokinin signalling, CHK receptors, nodulation, shoot development, leaf senescence

## Abstract

In this study, we expanded the understanding of cytokinin (CK) perception in legumes by generating and characterizing novel pea cytokinin receptor mutants carrying mutations for the four cytokinin histidine kinase *CHK* genes in pea, *CHK1*, *CHK2*, *CHK3* and *CHK4*. We constructed single, double, triple and quadruple mutants and analyzed their shoot, root, and nodulation phenotypes. We evaluated their contributions to the activation of CK-responsive genes, *TCSn* promoter activity, and used RNAi knockdowns of *CHK1* to explore its role in nodulation. We found key roles for CHK1 in promoting nodulation, CHK3 in delaying leaf senescence and CHK4 in promoting leaf size and axillary shoot branching. Traits such as stem elongation and width as well as shoot and root size were regulated redundantly by the CHK receptors. Overall, this work provides a genetic dissection of cytokinin receptor function in pea, advancing our understanding of hormone signaling in a crop legume and offering genetic insights with potential applications for improving both shoot architecture and symbiotic efficiency.

## Introduction

The plant hormone cytokinins (CKs) regulate a wide range of growth and developmental processes in plants. Much of our understanding of the genetic control of cytokinin biosynthesis, transport, perception, and response has been derived from studies with *Arabidopsis thaliana*, although more recently studies have focused in non-vascular plants, model grasses such as rice, and model legumes (for review see Argueso and Kieber, 2024). CKs are perceived via histidine kinase receptors (CHKs), that are encoded by relatively small gene families; 3 in *Arabidopsis*, 4 in rice, 2 in *Marchantia polymorpha*, and 4 in both legumes *Medicago truncatula* and *Lotus japonicus* (Held et al., 2014; Boivin et al., 2016; Rashotte, 2021). The perception of CK by CHKs initiates a canonical two-component signaling cascade: upon CK binding, CHKs autophosphorylate and transfer the phosphate to histidine phosphotransfer proteins (AHPs), which in turn activate type-B response regulators (RRBs) to induce transcription of CK-responsive genes (Tan et al., 2019). The expression of type-A RRs (RRAs) is induced in response to CK perception and provides a negative feedback to fine-tune CK response (To et al., 2004; To and Kieber, 2008). The CHK receptors have been shown to display different developmental roles in plants, likely in part due to unique expression patterns and different affinities for various species of cytokinins (Stolz et al., 2011; Choi et al., 2012; Boivin et al., 2016; Takeuchi et al., 2021).

In the model legumes *Lotus japonicus* and *Medicago truncatula*, the primary focus of studies with *chk* mutants has been on the impact of cytokinin perception on symbiotic relationship with nitrogen-fixing bacteria leading to root nodulation (Gonzalez-Rizzo et al., 2006; Murray et al., 2007; Tirichine et al., 2007; Plet et al., 2011; Ng et al., 2015; Van Zeijl et al., 2015; Kohlen et al., 2018; Velandia et al., 2022). However, the detailed analysis of the impact of CHK on other aspects of legume shoot and root development is lacking. As single cytokinin receptors often have only weak developmental phenotypes (Higuchi et al., 2004; Nishimura et al., 2004; Held et al., 2014), suggesting high levels of functional redundancy, it is key to examine higher order mutants. In this article, we outline the generation and functional characterization of a set of novel single and higher-order *chk* cytokinin receptor mutants in pea (*Pisum sativum*) and identify key roles for different CHKs in shoot growth and development, root architecture, and nodulation in this crop legume plant.

In vascular plants, CKs have been shown to be key regulators of both shoot and root development as well as of responses to biotic and abiotic stresses (Argueso and Kieber, 2024). The relative contribution of each CHK receptor to these processes has been investigated thoroughly in *Arabidopsis* and more recently in rice, which in addition to 4 CHK receptors also contains an unusual CK receptor (CHARK) that has a serine/threonine kinase activity (Halawa et al., 2021). CHK receptors in *Arabidopsis* and rice exhibit redundancy in their regulatory roles on shoot and root growth, with higher order mutants in both species exhibiting reduced shoot and root size (Higuchi et al., 2004; Nishimura et al., 2004; Riefler et al., 2006; Burr et al., 2020), likely as a result of reduced shoot and root apical meristem activity (Higuchi et al., 2004; Chun et al., 2023). More specific roles of some CHK receptors in specific shoot growth processes have been reported, such as *At*HK2 and *At*HK3 or *Os*HK5 and *Os*HK6 that redundantly promote stem in soybean elongation and leaf size (Nishimura et al., 2004; Riefler et al., 2006; Bartrina et al., 2017; Burr et al., 2020; Chun et al., 2023). In addition, *At*HK2 and *At*HK3 are required to suppress leaf senescence in response to exogenous CK and during development (Kim et al., 2006; Riefler et al., 2006), while this is mediated by *Os*HK4 in rice (Chun et al., 2023). A key role for CHKs in promoting vascular development in both shoots and roots was also revealed in *chk* mutants of *Arabidopsis* (*At*HK2, *At*HK3), *Medicago* (*Mt*CRE1), and rice (*Os*HK5 and *Os*HK6) (Nishimura et al., 2004; Hejatko et al., 2009; Laffont et al., 2015; Burr et al., 2020).

The roles of CHK receptors in regulating shoot and root branching is somewhat less clear. Exogenous CK application studies indicate that CK promotes the outgrowth of shoot axillary buds into shoot branches (Beveridge et al., 2023) and has an overall negative effect on the induction and elongation of lateral roots (Argueso and Kieber, 2024). No shoot branching phenotype of *Arabidopsis* single and double *chk* mutants has been reported (reviewed by Walker and Bennett, 2024), and it is difficult to distinguish if the reduced number of inflorescence branches of the *Atchk* triple mutant could be indirectly due to the very reduced shoot size and delayed bolting (Nishimura et al., 2004). In rice, several *Os*CHKs promote tillering, including a predominant role for *Os*HK4 (Yao et al., 2023) and a redundant role for *Os*HK5 and *Os*HK6 (Burr et al., 2020). Recent studies also indicate that CHKs regulate inflorescence architecture in rice panicles (Burr et al., 2020; Chun et al., 2023). Although, as outlined above, disruption of CHK receptors overall leads to a reduction in root size, both positive and negative effects of CK perception on root branching have been reported. In *Arabidopsis*, loss- and in some cases gain-of function mutants in *At*HK2, *At*HK3 and *At*HK4 suppress main and lateral root growth and restrict lateral root number (Riefler et al., 2006; Bartrina et al., 2017). Similarly, *Medicago MtCRE1* RNAi lines or *Mtcre1* mutant roots are insensitive to CK and form more lateral roots (Gonzalez-Rizzo et al., 2006; Laffont et al., 2015). In contrast, in *Lotus*, *Lj*HK1 and *Lj*HK3 act redundantly to promote lateral root number (Held et al., 2014).

Studies in legumes have identified that CK acting through CHK receptors are essential for nodulation (for review see Gamas et al., 2017; Velandia et al., 2022). In the model legumes studied, compatible rhizobia infect roots via root hairs, and invade the new root nodule organs that form mainly from the inner root cortex. One CHK receptor, encoded by *Mt*CRE1 in *Medicago* and the *Lj*LHK1 orthologue gene in *Lotus*, has a key role in promoting symbiotic nitrogen-fixing nodule organogenesis within the inner root cortex in response to CK. Loss of function mutations in *Mt*CRE1/*Lj*LHK1 demonstrated that these receptors promote local auxin accumulation, symbiotic gene expression, and cortical cell division leading to nodule organogenesis (Plet et al., 2011; Ng et al., 2015; Van Zeijl et al., 2015; Kohlen et al., 2018). Indeed, gain-of-function *cre1* and *lhk* mutants and exogenous CK induce nodule-like structures in the absence of rhizobia, demonstrating the central positive role of this CK perception system during nodule organogenesis (Tirichine et al., 2007; Hayashi et al., 2010; Madsen et al., 2010; Held et al., 2014; Boivin et al., 2016; Gauthier-Coles et al., 2019). Recently, a CRISPR mutant targeting *GmCRE1a/b/c/d* was also reported to form less nodules that have reduced nitrogen fixation capacity (Sun et al., 2023). In addition to CRE1, there is some level of redundancy with other CHK receptors in promoting nodule formation, as suppression of *MtHK2* and *MtHK3* expression via RNAi further suppressed nodule number in the *Medicago Mtcre1* mutant (Boivin et al., 2016). Similarly in *Lotus*, compared to the reduced nodule number observed in *Ljlhk1* mutants, a complete loss of nodule formation occurred in *Ljhk1 lk1a lhk3* triple mutants (Held et al., 2014). In addition, CK plays a negative role in regulating infection events in the epidermis in the determinate nodulator *Lotus*, acting via feedback mechanisms that may involve ethylene biosynthesis (Murray et al., 2007; Hansen et al., 2009; Held et al., 2014; Miri et al., 2016; Reid et al., 2016; Miri et al., 2019). However, studies with *Mtcre1* mutants and CK application suggest that in the indeterminate nodulators *Medicago* and pea, CK does not reduce the number of infection events, suggesting species-specific differences in the CK-mediated regulation of rhizobial infection (Lorteau et al., 2001; Gonzalez-Rizzo et al., 2006; Plet et al., 2011; Velandia et al., 2024).

Most studies of the effect of CKs on nodulation have focused on *M. truncatula* and *L. japonicus*, and only recently soybean for CRE1, while CHK functions in other crop legumes such as *P. sativum* remain underexplored. In addition, while cytokinin receptor functions in shoot and root development have been relatively well characterized in *Arabidopsis*, and more recently in rice, the roles of individual CK receptors in regulating shoot growth, leaf development and senescence, shoot branching and root development, have not been systematically investigated in any legume. Given the different roles for CK in regulating rhizobial infections between *Lotus* and *Medicago*, examining the role of CHK in another legume would be also informative. In this study, we expanded the understanding of cytokinin perception in legumes by generating and characterizing novel pea cytokinin receptor TILLING mutants carrying mutations in the four *CHK* genes in pea, *CHK1*, *CHK2*, *CHK3*, and *CHK4*. We generated single, double, triple, and quadruple mutants and analyzed their cytokinin response, as well as their shoot, root, and nodulation phenotypes.

## Methods

### Plant material, growing conditions and shoot phenotyping

Single and double mutants for *CHK* receptor genes in *Pisum sativum* L. (Cameor cultivar) — *Ps*chk1-1422 (*Psat7g004720*), *Ps*chk2-3764 (*Psat7g077280*), *Ps*chk3-2199 (*Psat5g097280*), and *Ps*chk4-4524 (*Psat2g004360*) — were selected from reverse genetic screens carried out in an Ethane Methane Sulfonate (EMS)-mutagenized Targeting Induced Local Lesions IN Genomes (TILLING) population in the Cameor genotype (Dalmais et al., 2008). The mutation detection was achieved by MiSeq Illumina sequencing of PCR-amplified ~500bp targeted regions on 2500 M2 mutant families. Following the identification of mutant alleles for each receptor, plants underwent two rounds of backcrossing to the Cameor wild-type. Double mutants were generated through crossing and subsequently backcrossed once. Each *chk* mutation originated from an independent EMS-derived TILLING line. The generation of double, triple, and quadruple mutants involved multiple rounds of genetic crossing and segregation between independently mutagenized genomes, which is expected to efficiently dilute unlinked background mutations. The presence of mutations in each of the four *chk* genes was confirmed by sequencing and restriction fragment length polymorphism (RFLP) genotyping (**Supplementary Table 1** and **Supplementary Figure 1**).

Unless stated otherwise, pea plants were cultivated in a glasshouse under an 18-hour photoperiod. Temperature ranged from 13–21 °C during cooler months and 17–35 °C during warmer months. Plants were grown in 2 L pots containing a 1:1 mixture of vermiculite and gravel, topped with potting mix. Seeds were nicked prior to sowing, and two seeds were planted per pot. Plants were watered three times per week, and Peters Professional nutrient solution (ICL, Australia) was applied weekly.

For shoot phenotype measurements, plants were grown under a 12-hour photoperiod under glasshouse conditions. Eight weeks after planting, shoot length between nodes 3 and 7, first flowering node, number of expanded leaves, number of branches, branch length, and branching nodes were recorded. For quadruple and single mutants, leaflet area (at node 11), as well as shoot width, were also measured. For the senescence analysis, plants were grown 20 °C day/17 °C night with 18 hours of light, and pictures of the leaflet at the fourth node were taken weekly from week 2 after planting. Images were analyzed using an automated colorimetric assay adapting the method described by Bresson et al. (2017) with the R code available at https://github.com/alextomkinson/Leaf-Senescence-Colorimetry. In summary, leaf senescence progression was quantified by calculating the percentage of green, yellow, brown, and white pixels for each leaflet at each time point. Statistical analyses focused on the accumulation of brown pixels as a quantitative measure of senescence progression. Data were analyzed using linear mixed-effects models.

For nodulation experiments, twelve seeds per genotype were surface-sterilized in 70% (v/v) ethanol for 1 min, followed by five rinses with sterile distilled water. Seeds were sown in 2 L pots containing sterile vermiculite and grown in a controlled-environment growth cabinet under an 18 h light/6 h dark photoperiod, with day/night temperatures of 20 °C/15 °C. Plants were irrigated three times per week with sterile water and supplied weekly with a nitrogen-free modified LANS nutrient solution (Hewitt, 1966). The *Rhizobium leguminosarum* symbiovar *viciae* strain RLV248, carrying the plasmid pHC60 for constitutive GFP expression (RLV248G) (Cheng and Walker, 1998) was cultured in Yeast Mannitol Broth (YMB) supplemented with 10 μg mL^−1^ tetracycline at 25 °C for 3 days with shaking at 170 rpm.

Seven days after planting, each pot was inoculated with 50 mL of a 1:10 dilution of the bacterial culture. Plants were harvested 28 days after planting, and phenotypic parameters including number of expanded leaves, number of branches longer than 1 cm, and shoot fresh and dry weights, were recorded. The three uppermost intact lateral roots were dissected and fixed in 1% (w/v) paraformaldehyde (PFA) for subsequent scoring of infection threads using fluorescence microscopy. Remaining roots were stored in ethanol for total nodule counting and for determination of root and nodule dry weights.

### Genotyping

For DNA extraction, fresh tissue from a young leaf was collected in liquid nitrogen and stored at -80 °C for subsequent analysis. DNA extraction was carried out using the cetyltrimethylammonium bromide (CTAB) method, resuspended in DNAse free water, and quantified using a spectrophotometer. Genotyping of *chk* mutants was performed using restriction fragment length polymorphism (RFLP) markers. For each mutation, PCR primers were designed to amplify a region flanking the EMS-induced nucleotide substitution. The mutation either created or abolished a restriction enzyme recognition site, allowing discrimination between wild-type and mutant alleles following enzymatic digestion. Homozygous wild-type and mutant individuals were identified based on the presence or absence of the expected restriction fragments, while heterozygous plants displayed both digested and undigested PCR products (**Supplementary Figure 1**). The specific primers, restriction enzymes, and expected digestion patterns for each *CHK* gene are listed in **Supplementary Table 1**.

### Phylogenetic analysis of cytokinin receptors and type A response regulators in pea and evaluation of amino acid substitutions in the mutants

The reciprocal best hit approach was used to identify orthologues for *CHK* receptors and *RRA* genes in pea. Sequences of *CHK* and *RRA* from *A. thaliana* and *M. truncatula* were used as queries (Tan et al., 2019) to search for the closest orthologues within the pea ‘Cameor’ genome (https://urgi.versailles.inra.fr/Species/Pisum/Pea-Genome-project). Identified pea genes were then subjected to a reciprocal BLAST search against the *A. thaliana* TAIR10 genome and the *M. truncatula* Mt4.0v1 genome (https://phytozome-next.jgi.doe.gov). The *Marchantia polymorpha* CHK2 gene sequence was retrieved from Phytozome.

Gene sequences were aligned using the Muscle algorithm within the MEGA software version 10.2.6 (Tamura et al., 2021), with default options. The aligned sequences were then used to construct a phylogenetic tree with the Maximum Likelihood Method in the MEGA software (Tamura et al., 2021), with default settings. The impact of amino acid substitutions was assessed using a Sequence-homology-based sorting Intolerant From Tolerant (SIFT) analysis conducted for each *chk* mutant allele (https://sift.bii.a-star.edu.sg).

### Application of synthetic cytokinin 6-BAP in wild type and *chk* quadruple mutants

Pea seeds were planted in 2 liter pots filled with vermiculite. Wild-type and quadruple *chk1chk2chk3chk4* mutant plants were grown in a growth cabinet with 18 hours of light, at 20 °C during the day and 15 °C at night and were watered three times a week. On day 6, pea plants were carefully removed from the pots, and the entire root system was submerged in a water solution containing 100μM 6-Benzylaminopurine (6-BAP) or water alone for the control treatments. For leaf tissue, on day 14 after planting, the youngest leaf was infiltrated with 50μM BAP or water alone as a control. Treated root and leaf tissue was collected 1 or 2 hours after BAP application for RNA extraction.

### RNA extraction and RT-qPCR analysis

Root RNA was extracted using the ISOLATE II RNA Mini Kit (Bioline) following the manufacturer’s instructions. RNA quality and concentration were measured by absorbance at 260nm using a Nanodrop spectrophotometer (Thermo Scientific). cDNA was synthesized from 1μg of RNA using the SensiFAST cDNA Synthesis Kit (Bioline).

Reverse transcription quantitative PCR (RT-qPCR) was performed using a Rotor-Gene Q 2-PLEX (Qiagen) with the SensiMix™ SYBR^®^ No-ROX Kit (Bioline), in duplicates for each sample. The transcription initiation factor IIA (*TFIIa*) or *Actin* 7 genes were used as housekeeping genes to calculate relative expression levels. The list of primers used for qPCR is provided in the **Supplementary Table 2**.

### Hairy root transformation of wild-type and *chk* quadruple mutants

The *Agrobacterium rhizogenes* strain Arqua1 carrying the plasmid *PCR1::TCSn::GUS* was used for pea root transformation of wild-type Cameor and quadruple mutant plants according to Velandia et al. (2024). Upon transferring into pots, plants were inoculated with the GFP-tagged rhizobia strain RLV248G. Harvesting was done four weeks post rhizobial inoculation.

Individual roots were screened for transformation using DsRed as a marker for transformation. One transformed root per plant was collected for RNA extraction. The remaining transformed roots were stained for β-glucuronidase and subsequently stored in 1% paraformaldehyde. Counting of nodules and infection threads was performed using a fluorescence microscope (ZEISS Axioscope 5). We observed nodulation structures (infections, developing nodules, mature nodules) using the GFP-tagged bacteria to identify infections and developing nodules. We then recorded if each structure was associated with GUS staining (blue, GUS-positive) or it was not (GUS-negative) by observing same root section in brightfield.

### RNA interference silencing of the *CHK1* gene

Hairpin RNA constructs were used to silence the *CHK1* gene in wild-type roots. A target region of 391bp located in the Cyclase/Histidine Kinase Associated Sensory Extracellular (CHASE) domain (489 to 880bp of CDS) (*RNAi::CHK1_CHASE_*), and a region of 380 nucleotides in the histidine kinase (HIS) domain (1200-1500bp CDS) (*RNAi::CHK1_HIS_*), were selected as inverted repeat fragments. These fragments were incorporated into the pRNAi-GG construct (Yan et al., 2012), modified to express the DsRed as a transformation marker. *A. rhizogenes*-mediated root transformation was performed as described by Velandia et al. (2024). The nodulation phenotype was evaluated in roots harvested 3 and 5 weeks post-rhizobium inoculation. Transformed and untransformed roots were collected for quantitative RT-PCR analysis of *CHK* receptor genes using primers listed in **Supplementary Table 2**.

## Results

### The pea *Ps*CHK receptor family and predicted effects of *chk* mutations identified by TILLING

**Figure 1:**
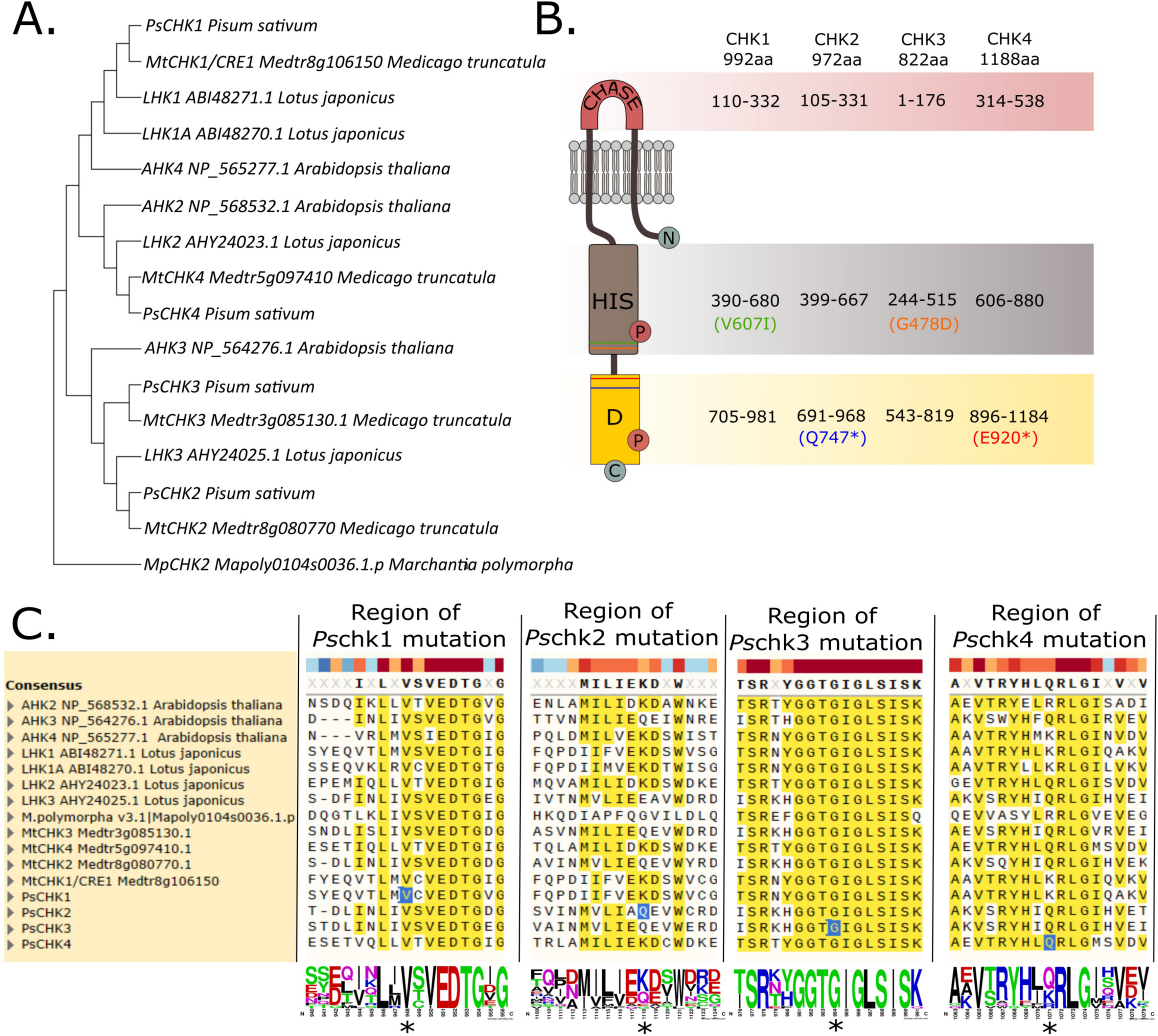
Phylogenetic and structural characterization of CHK cytokinin receptors and mutation sites identified in the pea mutants. **(A)** Phylogenetic tree of CHK receptor family members from *Arabidopsis thaliana*, *Medicago truncatula*, *Lotus japonicus*, and *Pisum sativum*, with *Marchantia polymorpha* MpCHK2 used as an outgroup. **(B)** Schematic representation of the canonical structure of a histidine kinase cytokinin receptor (CHK), showing the main domains and the amino acid ranges corresponding to each domain in the four *Pisum sativum* CHK receptors, as determined by InterProScan. Mutations in each receptor are indicated in distinct colors and represented using single-letter amino acid codes in parentheses (* indicates a stop codon). The relative position of each mutation within the CHK receptor is indicated by a horizontal line. Domain abbreviations: CHASE, Cyclase/Histidine Kinase Associated Sensory Extracellular domain; HIS, Histidine Kinase domain; D, Receiver domain; C, carboxyl terminus; N, amino terminus; P, phosphoryl group. **(C)** Alignment of the mutated regions in each pea CHK receptor, with sequence logos showing amino acid conservation across species. Mutated residues in pea chk mutants are highlighted with a blue background and marked with an asterisk. Alignment parameters: gap opening = 4, gap extension = 0, cluster method = UPGMA, iterations = 16. Phylogenetic tree parameters: 1000 bootstrap replicates, Tamura–Nei model, uniform rates, and Nearest Neighbor Interchange heuristic method.

A search for orthologues of *CHK* genes in pea revealed the presence of four putative genes within the pea CHK family *PsCHK1, PsCHK2, PsCHK3* and *PsCHK4* (**Figure 1**). *PsCHK1* is most closely related to *MtCHK1*/*CRE1*, *LjLHK1/1A* and *AtHK4. PsCHK4* is most closely related to *MtCHK4, LjLHK2* and *AtHK2*, while in a third clade containing *AtHK3* and *LjHK3*, *PsCHK3* is closely related to *MtCHK3* and *PsCHK2* to *MtCHK2* (**Figure 1A**). The tissue specific expression of these receptors was examined using previously published RNAseq data (**Supplementary Table 3**; Alves-Carvalho et al., 2015). The four CHK receptors are expressed to some extent across all tissues examined. *PsCHK1* is more highly expressed in stems, peduncles, and mature nodules, *PsCHK2* has its highest expression in developing nodules, *PsCHK3* is expressed in most shoot and root tissues, and *PsCHK4* is expressed throughout the plant.

Mutant alleles for each *CHK* receptor gene were identified by TILLING from an EMS population in the pea cv. Cameor background. Analysis of the mutant alleles revealed that *chk1* and *chk3* have substitutions in a predicted conserved amino acid of the histidine domain (**Figures 1B, C**). The *chk1* allele carries a valine-to-isoleucine substitution at position 607, whereas *chk3* features a glycine-to-aspartic acid substitution at position 478 (**Figures 1B, C**). A SIFT analysis assessing the potential impact of these substitutions on receptor function indicated that the *chk1* allele may be tolerated and retain partial or complete activity, likely because a hydrophobic amino acid is replaced by another hydrophobic residue. Conversely, the *chk3* allele was predicted to be deleterious to the function, as the mutation occurs in a highly conserved amino acid and involves a substitution from a neutral to a negatively charged residue (**Figures 1B, C**). On the other hand, the *chk2* and *chk4* mutant alleles resulted in truncations within the receiver domain of the protein (**Figures 1B, C**). Sequence alignment with *Arabidopsis* cytokinin receptors indicates that the premature stop codons in *chk2* and *chk4* occur upstream of the conserved AHK4 Asp973 residue that serves as the phosphorylation acceptor in the receiver domain, a residue shown to be essential for receptor function in *Arabidopsis*. These truncations therefore likely eliminate the entire functional receiver domain, predicting that *chk2* and *chk4* represent loss-of-function alleles (Hwang and Sheen, 2001; Inoue et al., 2001). The identification of three severely disrupted alleles and one mildly affected allele within the CHK receptor family prompted a detailed investigation of the phenotypic consequences associated with these *chk* receptor mutants. As previous studies have shown that CHK receptors function redundantly in regulating diverse developmental processes (e.g. Müller, 2011), to assess potential redundancy among pea CHK receptors, a quadruple mutant carrying mutations in all four *chk* genes was generated.

### *TCSn* cytokinin responsive promoter activation during nodulation is altered in the *chk1 chk2 chk3 chk4* pea quadruple mutant

**Figure 2:**
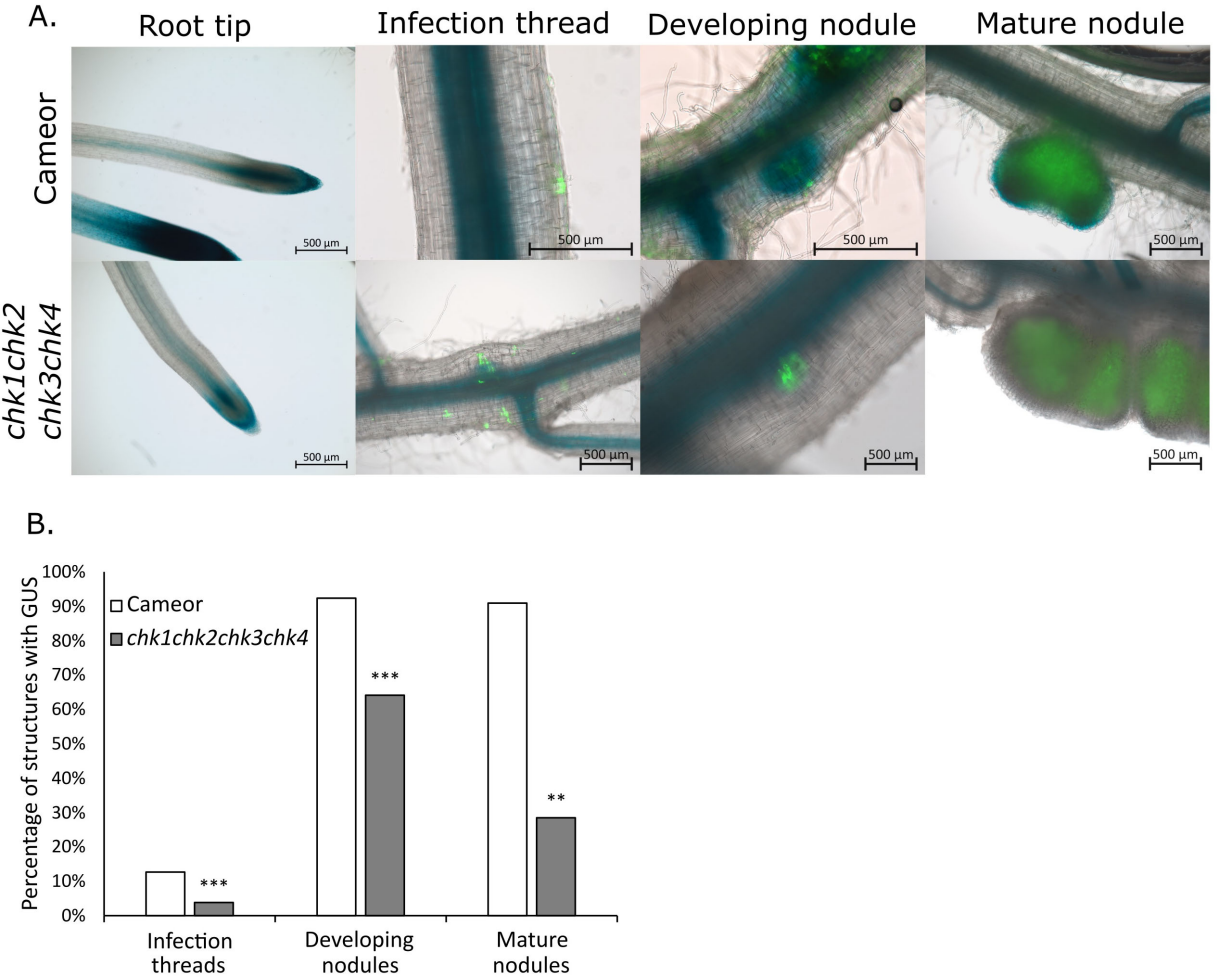
Altered cytokinin response patterns during nodulation in chk quadruple mutants. **(A)** Merged brightfield and fluorescence images showing root tips and nodules, where rhizobia were tagged with green fluorescent protein, in wild-type and chk quadruple mutant roots transformed with TCSn::GUS. GUS activity is shown in blue. Root tip correspond to lateral roots. **(B)** Percentage of infection threads (wild-type = 563, quadruple mutant n= 511), developing nodules (wild-type n= 93, quadruple mutant n= 53), and mature nodules (wild-type n= 11, quadruple mutant n= 14) associated with GUS activity in wild type and quadruple mutant. Asterisks indicate significant genotype × GUS activity interactions (2×2 chi-square test; **P < 0.01, ***P < 0.001).

The activation of cytokinin signaling in symbiotic nodulating roots was evaluated to assess the CK response of the *chk* quadruple mutant. CK plays a pivotal role in nodulation, and CHK receptor–dependent CK signaling activation is necessary and sufficient to induce nodule organogenesis (Tirichine et al., 2007; Plet et al., 2011; Van Zeijl et al., 2015). In pea, the *TCSn* cytokinin-responsive promoter was previously shown to be activated in developing and mature nodules (Velandia et al., 2024). To examine the CK response activation during nodulation, roots of wild-type and *chk* quadruple mutant plants expressing the *TCSn::GUS* cytokinin-responsive reporter were inoculated with rhizobia. Macroscopic examination of whole roots showed expression of the *TCSn::GUS* reporter in both wild-type and quadruple mutant plants (**Supplementary Figure 3**). However, closer microscopic analysis of nodulation structures revealed a reduced cytokinin response during nodule formation in the quadruple mutant, while lateral root tips had a similar pattern of *TCSn* activation in wild type and quadruple mutants (**Figures 2A, B**). The number of infection threads, developing nodules, and mature nodules exhibiting *TCSn* promoter activity was indeed significantly lower in quadruple mutants compared to wild-type plants, although promoter activity in root tips and vasculature was comparable between these two genotypes (**Figure 2B**).

We further examined whether the *chk* quadruple mutant retained responsiveness to exogenous cytokinin by analyzing the induction of *RRA* gene expression in leaf and root tissues. Eight pea orthologs of *M. truncatula RRA* genes were identified in the pea genome (**Supplementary Figure 2A**), and their expression was used as a molecular readout of cytokinin response activation (**Supplementary Figure 2B**). In both wild-type and quadruple mutant plants, *RRA2, RRA4, RRA8*, and *RR9* were significantly upregulated in roots following cytokinin application relative to mock-treated controls, with no significant differences in induction levels between genotypes (**Supplementary Figure 2B**). Similarly, *RRA2, RR3*, and *RRA8* showed increased expression in leaves of both genotypes after cytokinin treatment, again with comparable magnitudes of induction (**Supplementary Figure 2B**).

Interestingly, *RRA5* and *RR9* were downregulated in leaves in response to cytokinin, indicating that RRA responses are tissue-specific and do not always involve transcriptional activation. In the quadruple mutant, several *RRA* genes exhibited altered basal expression levels or modified responses to cytokinin. For example, *RRA4* failed to be induced in leaves, *RR6* showed increased basal expression in leaves but reduced basal expression in roots compared with the wild type, and *RR7* displayed higher basal expression and an opposite response to cytokinin in leaves relative to the wild type.

### *chk* pea mutants have an altered shoot development

**Figure 3:**
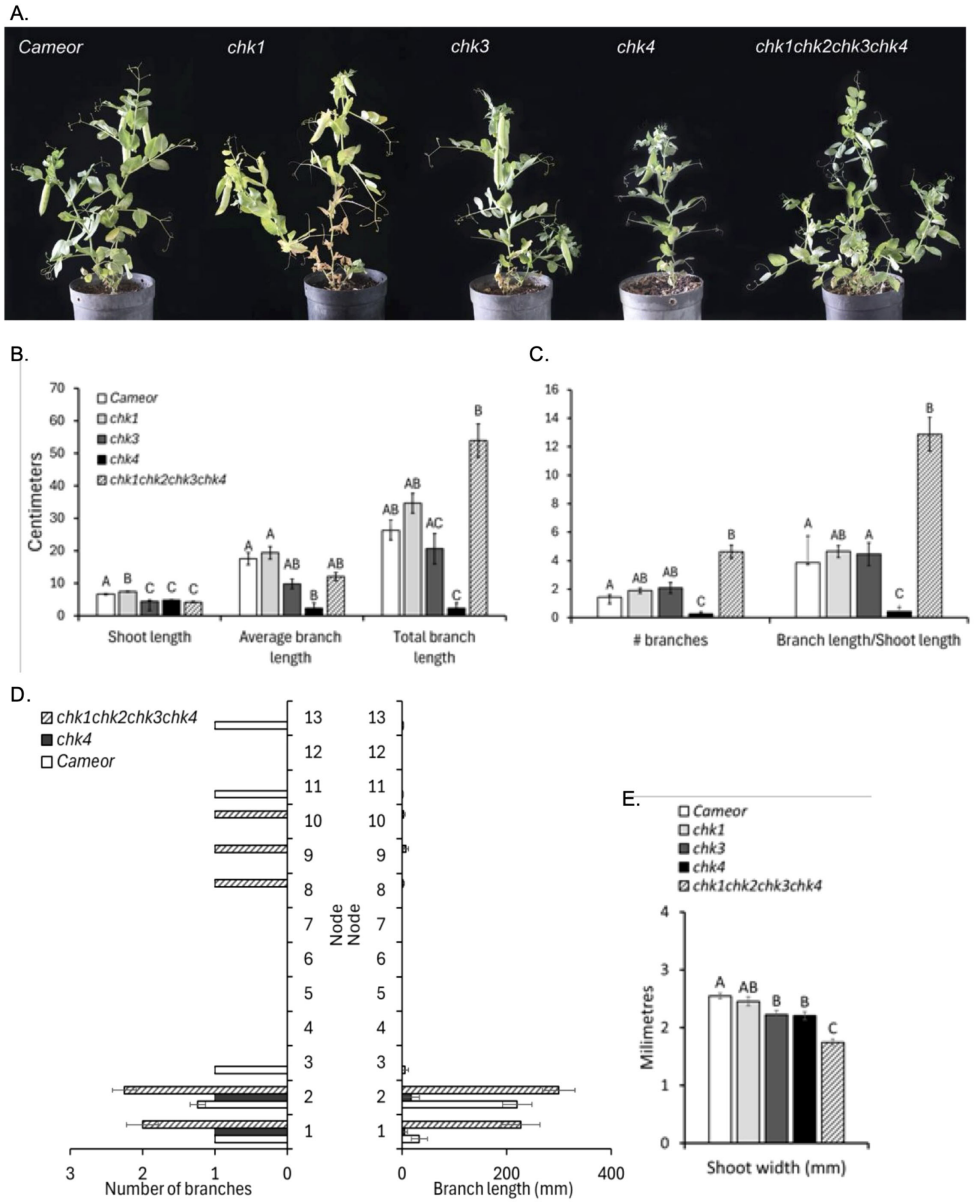
Shoot phenotype in chk single and quadruple mutants. **(A)** Representative shoot phenotypes of wild-type (WT), single *chk1*, *chk3*, *chk4*, and quadruple *chk1chk2chk3chk4* mutants. Plants were grown for 8 weeks after planting. **(B, C)** Quantitative analysis of shoot traits for WT, single mutants, and the quadruple mutant: shoot length between nodes 1–6, average branch length, total branch length, number of branches, and branch length relative to shoot length (WT and single mutants: n = 6; quadruple mutant: n = 10). **(D)** Branching and flowering node pattern of WT and quadruple mutant plants, showing node number (red circles), branch number and length per node. **(E)** Stem width for wild type, singles and quadruples. The asterisk indicates the node of first flowering. Bars represent means ± standard error. Different letters indicate statistically significant differences within a parameter (p < 0.05), as determined by one-way ANOVA with Tukey’s *post hoc* test or Kruskal–Wallis test with Bonferroni correction.

**Figure 4:**
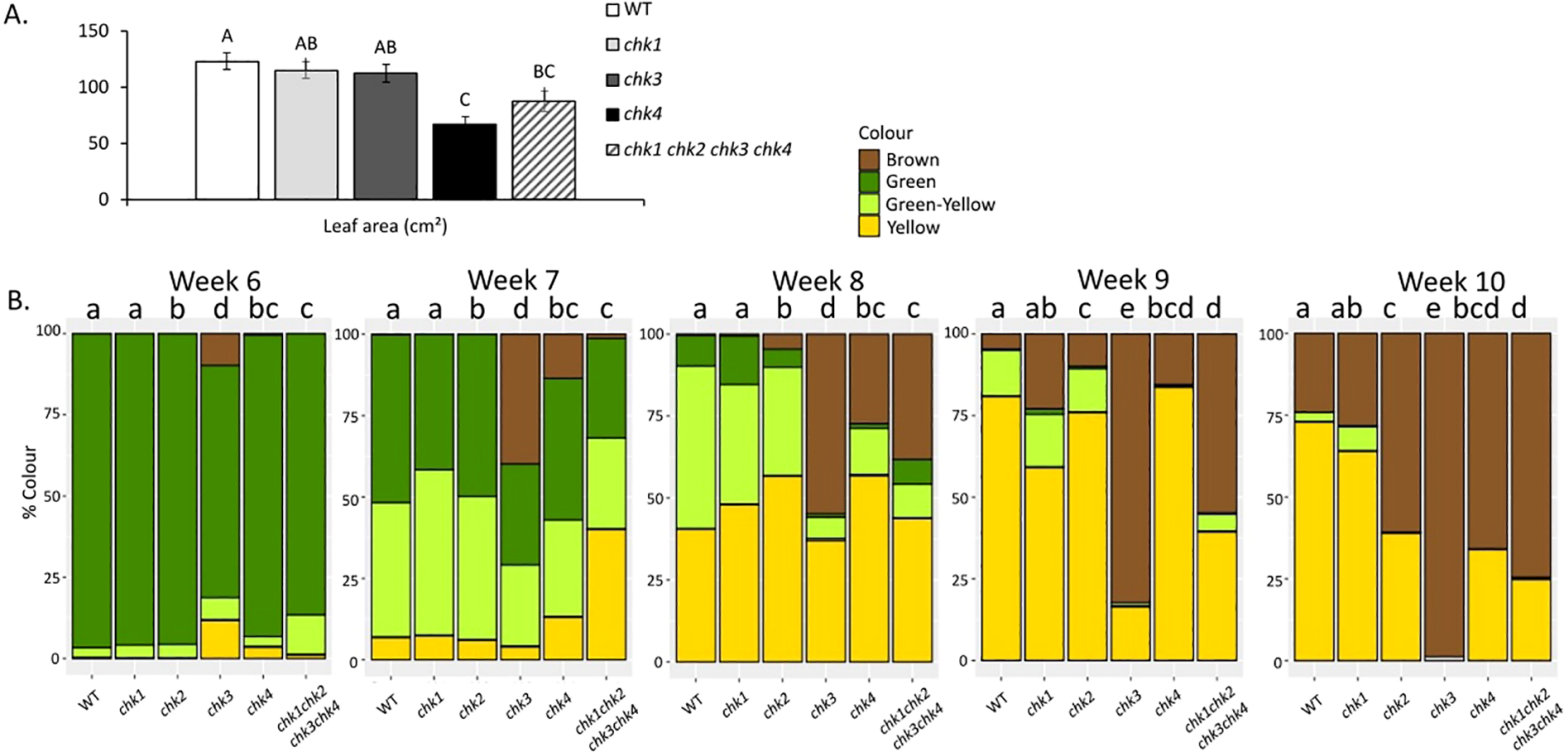
Leaflet size and senescence in chk single and quadruple mutants. **(A)** Leaflet area wild-type (WT) and quadruple *chk1chk2chk3chk4* mutants. Different letters indicate statistically significant differences within a parameter (p < 0.05), as determined by one-way ANOVA with Tukey’s *post hoc* test or Kruskal–Wallis test with Bonferroni correction. **(B)** Leaf color progression over time in leaflet 4 of WT, single *chk1*, *chk2*, *chk3*, *chk4*, and quadruple *chk1 chk2 chk3 chk4* mutants. The same leaf was imaged weekly from 6 to 10 weeks after planting. Senescence progression was analyzed using a linear mixed-effects model with genotype, time, and their interaction as fixed effects and plant identity as a random effect. Significant effects were detected for genotype (F_5,401_ = 25.5, *p* < 0.001), time (F_1,404_ = 340.3, *p* < 0.001), and genotype × time interaction (F_5,402_ = 16.1, *p* < 0.001). Different letters indicate significant differences among genotypes within the same week based on estimated marginal means with Šidák-adjusted *post hoc* comparisons (*p* < 0.05).

The contribution of single and combined CHK receptor genes on shoot development were analyzed using the different *chk* mutants. Shoot length was significantly affected by the different CHK mutations. Single mutant *chk3* and *chk4* plants displayed a significant reduction in shoot length (**Figures 3A, B**), with a similar decrease in shoot length in quadruple mutants compared to wild-type plants (**Figures 3A, B**). An independent experiment also revealed that *chk2* mutants had a small but significant reduction in shoot length, compared to the wild-type (**Supplementary Figure 4**). Similarly, the *chk3* and *chk4* double mutant, but not *chk1* or *chk2* single mutants, had significantly thinner stems than wild-type (**Figure 3E**; **Supplementary Figure 4D**), and this phenotype was even more pronounced in quadruple mutant plants (**Figure 3E**). Single *chk4* mutant plants displayed a significant reduction in leaflet area compared to wild-type and *chk1* and *chk3* mutants (**Figure 4A**), whereas *chk2* mutants did not display any difference in leaf area (**Supplementary Figure 4D**). A similar reduction in leaf area was observed in the quadruple mutants (**Figure 4A**), indicating that CHK4 is the major receptor regulating leaf area. The first flowering node was not affected by *chk* single or quadruple mutations (**Supplementary Figure 5**). Collectively, these results indicate that CHK2, CHK3 and CHK4 promote stem elongation, while CHK receptors act redundantly to regulate stem width and CHK4 promotes leaf area.

Nodulated plants were analyzed for their shoot and root dry weight, as well as for their lateral root number. There was no consistent effect in *chk* single or double mutant plants on overall shoot dry weight (**Supplementary Figures 6A, B**, **7A, B**), whereas all triple mutants and the quadruple mutant displayed significant reduction in shoot weight compared to the wild-type (**Supplementary Figures 6B**, **7**-**9**), suggesting that as reported in other species, there is a high level of functional redundancy between CHK receptors to control this trait in pea. There was no consistent effect of *chk* single, double, or triple mutations on root dry weight (**Supplementary Figures 6**-**9**), although the quadruple mutant displays a significant reduction in root dry weight compared to wild-type and single mutant plants (**Supplementary Figure 6**), again suggesting that many or all of the *CHK*s act to promote root growth. There was no consistent impact of any lower or higher order *chk* mutant combinations on lateral root number (**Supplementary Figure 6**-**9**).

CK is known to play a crucial role in the activation of axillary bud growth and shoot branching (Beveridge et al., 2023), but the impact of *chk* mutations in branching of dicots has not been reported outside of *Arabidopsis*. Given the strong link between branching and flowering transition in *Arabidopsis*, examining the role of CHKs in a species like pea that display vegetative branching is essential for a broader understanding of CHK regulation of shoot architecture. Branch number and length per node, total branch length, and the ratio of branch length to total shoot length, were examined (**Figures 3B–D**). Wild-type plants produced some basal and aerial branches (**Figures 3C, D**). The *chk4* single mutant displayed almost no branching, with a significant reduction in the number of branches and in the length of branches compared to the wild-type (**Figures 3B–D**). These phenotypes resulted in a significant reduction of the ratio of the branch length to the main shoot length in this *chk4* mutant (**Figure 3C**). No significant change in branching was observed in *chk1* or *chk3* mutants (**Figures 3B–D**), or in *chk2* in an independent experiment (**Supplementary Figure 4**). In contrast, the quadruple mutant produced significantly more shoot branches than the wild-type, and these branches were significantly longer (**Figures 3A–D**). The branch-to-shoot length ratio was also significantly increased in the quadruple mutant relative to the wild-type (**Figure 3C**), indicating a possible functional redundancy of CHK2 and CHK3 receptors in suppressing branching.

CK signaling can delay leaf senescence in detached leaflets, as reported in different species, and several *chk* mutants have been previously shown to display early leaf senescence (Kim et al., 2006; Chun et al., 2023). The impact of *chk* mutations on natural leaf senescence is however less well understood. Pea leaflets were imaged every week over 8 weeks from 2 weeks after planted, and their change in color was analyzed (**Figure 4**; **Supplementary Figure 10**). Quantitative analysis of leaf senescence revealed strong effect of genotype and time on the accumulation of brown pixels, with significant genotype × time interaction, indicating that senescence progressed at different rates among genotypes. In particular, *chk2, chk3, chk4*, and the quadruple mutant displayed accelerated senescence compared to wild type. Among the single mutants, *chk3* showed the strongest phenotype, with a pronounced early onset of leaf senescence by 6 weeks after planting, as indicated by the increase in the percentage of leaves classified as yellow and brown. In contrast, wild-type plants did not display brown coloration until 9 weeks after planting. By 9 weeks, *chk3* mutant leaves were almost entirely brown, while wild-type leaves were less than 25% brown at this time. This early onset of senescence was not due to any change in node of first flower (**Supplementary Figure 5**). The quadruple mutant exhibited a similarly strong early-senescence phenotype, whereas senescence acceleration was more moderate in *chk2* and *chk4* and absent in *chk1* (**Figure 4**; **Supplementary Figure 10**). Together, these results indicate that *PsCHK3* plays a central role in delaying natural leaf senescence, with additional, partially redundant contributions from other CHK receptors.

### CHK1 is the main receptor regulating nodulation in pea

CHK receptors are known to mediate cytokinin signaling during nodulation, playing a key role in nodule formation while also participating in negative feedback to control rhizobial infection at least in *Lotus* (Plet et al., 2011; Van Zeijl et al., 2015; Miri et al., 2019). The nodulation phenotype of *chk* single, double, triple and quadruple mutants including nodule number and infection thread numbers was analyzed using GFP-tagged rhizobia, and shoot and root phenotypes were also analyzed and are discussed above.

**Figure 5:**
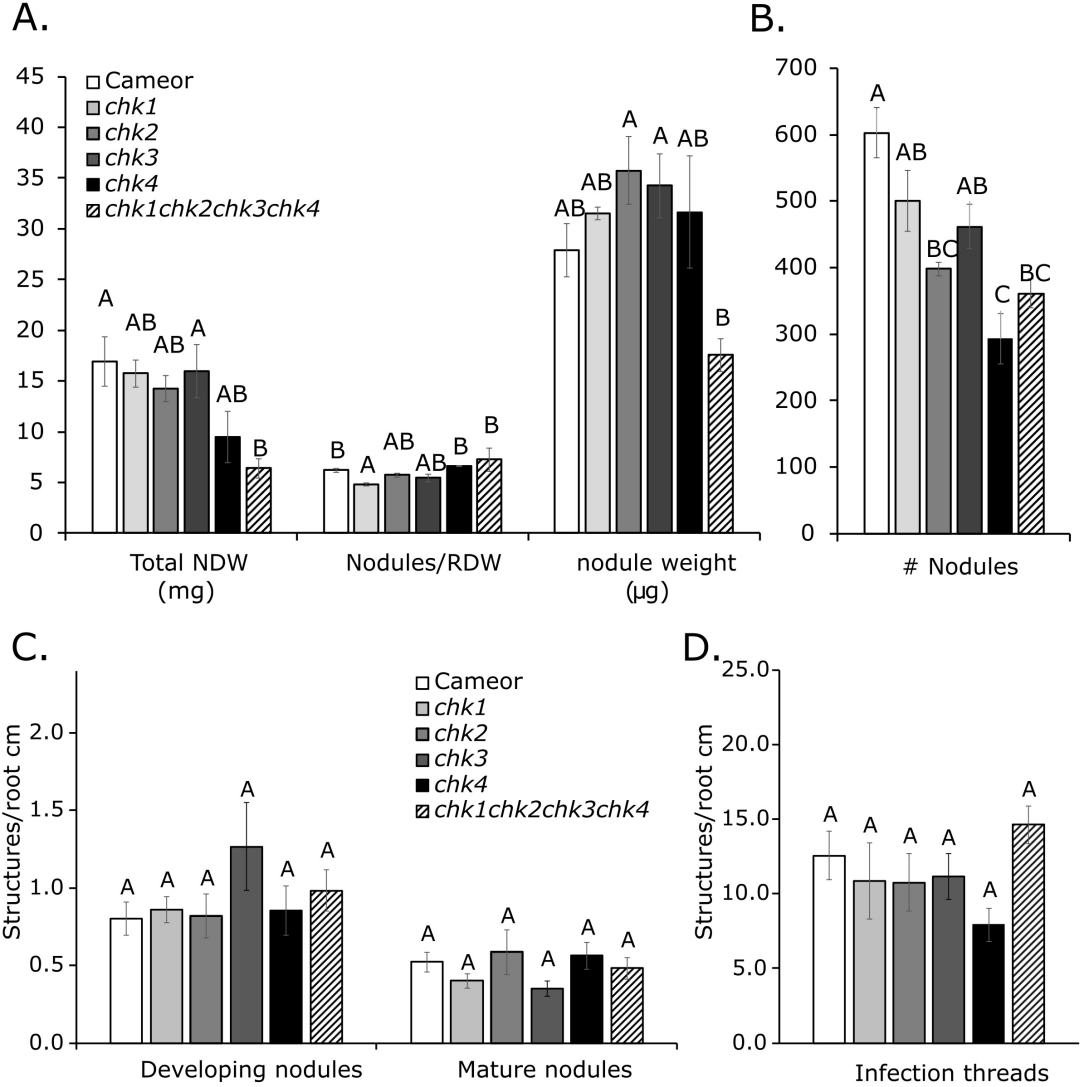
Nodulation phenotype of *chk1*, *chk2*, *chk3*, *chk4* single mutants and the *chk1chk2chk3chk4* quadruple mutant. **(A)** Total nodule dry weight (Total NDW) (ANOVA, p = 0.0151; n = 3), number of nodules normalized to root dry weight (Nodules/RDW) (ANOVA, p = 0.00657; n = 3), and average individual nodule weight (ANOVA, p = 0.0192; n = 3). **(B)** Total number of nodules per plant (#Nodules) (ANOVA, p = 0.0000407; n = 3). **(C, D)** Number of structures per centimeter of root for **(C)** developing and mature nodules (ANOVA, p = 0.447 and p = 0.417, respectively) and **(D)** infection threads (ANOVA, p = 0.149), counted under a fluorescence microscope using GFP-tagged rhizobia. For **(C, D)**: *chk1*, *chk3*, and *chk4* (n = 6); *chk2* (n = 4); *chk1 chk2 chk3 chk4* mutants, and wild-type (WT), one root per plant (n = 12). Bars represent means ± standard error. Different letters indicate statistically significant differences between genotypes within a trait (*p* < 0.05), as determined by one-way ANOVA followed by Tukey’s *post hoc* test or Kruskal–Wallis test with Bonferroni correction.

Quantification of total nodule number per plant revealed that the single mutants *chk2* and *chk4*, as well as the *chk1 chk2 chk4* triple mutant and the quadruple mutant, exhibited a significant reduction in nodule number compared with the wild-type, with no significant changes in other genotypes (**Figure 5**; **Supplementary Figures 6**-**8**). However, due to the lower root dry weight observed in these mutants, only the *chk1* single mutant showed a significant decrease in nodule number per gram of root dry weight compared with the wild-type (**Figure 5**; **Supplementary Figures 7**-**9**). Although the total nodule dry weight per plant was significantly lower in the quadruple mutant relative to the wild-type, when combined with the observed lower nodule number per plant, this did not lead to any significant reduction in the average nodule weight (**Figure 5A**). None of the other mutant combinations showed significant differences in total nodule dry weight per plant or in individual nodule weight (**Figure 5**; **Supplementary Figures 7**-**9**). To further examine the impact of *chk* mutations across different stages of nodulation, we quantified the number of infections, developing and mature nodules, per centimeter of root using GFP-tagged rhizobia. The numbers of infection threads, developing nodules, and mature nodules, per root length did not differ significantly among any of the mutant combinations and the wild-type (**Figure 5**; **Supplementary Figures 7**-**9**). These results indicate that the *chk* mutations examined did not substantially affect the frequency of epidermal infection events or the number of nodule primordia.

**Figure 6:**
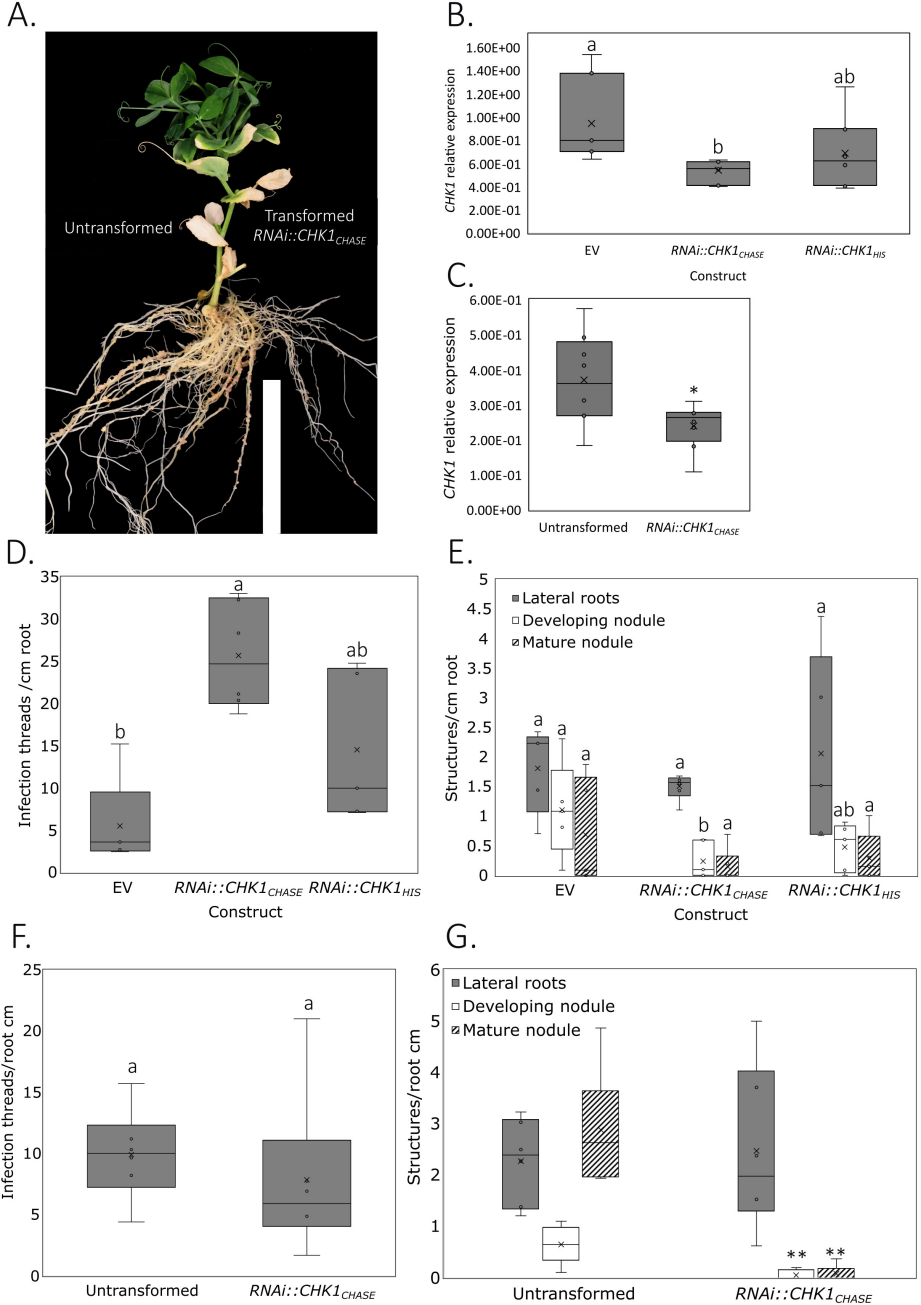
A knockdown of the CHK1 receptor impairs nodulation in pea roots. **(A)** Representative image of a pea plant transformed with *Agrobacterium rhizogenes* carrying the *RNAi::CHK1_CHASE_* construct, showing transformed roots (right) and untransformed roots (left). **(B, C)** Relative expression of *CHK1* in roots transformed with **(B)** empty vector (EV), *RNAi::CHK1_CHASE_*, or *RNAi::CHK1_HIS_* constructs (ANOVA, *p* < 0.05; *n* = 8), and **(C)** *RNAi::CHK1_CHASE_*-transformed versus untransformed roots (t-test, *p* = 0.03; *n* = 8). Expression values were normalized to the geometric mean of reference genes *Actin7* and *TFIIa*. **(D, E)** Quantification of nodulation structures per centimeter of root at 3 weeks post inoculation with GFP-tagged rhizobia for roots transformed with EV, *RNAi::CHK1_CHASE_*, and *RNAi::CHK1_HIS_* constructs: **(D)** infection threads (ANOVA, *p* = 0.0014) and **(E)** developing (ANOVA, *p* = 0.049) and mature nodules (ANOVA, *p* = 0.33; *n* = 5). **(F, G)** Quantification of nodulation structures per centimeter of root at 5 weeks post inoculation in *RNAi::CHK1_CHASE_*-transformed and untransformed roots: **(F)** infection threads (t-test, *p* = 0.53) and **(G)** developing (t-test, *p* = 0.009) and mature nodules (t-test, *p* = 0.0016; *n* = 6). Box plots show the median, interquartile range, and individual data points. Grouping letters indicate statistically significant differences (*p* < 0.05) between genotypes within a trait, determined by one-way ANOVA with Tukey’s *post hoc* test. Asterisks indicate significance levels for t-tests: *p* < 0.05 (*), *p* < 0.01 (**).

Studies in the model legumes *Lotus japonicus* and *Medicago truncatula* have identified CHK1 as the main CK receptor involved in nodulation. As we expected only a mild predicted impact of the pea *chk1* allele on receptor function, and we observed no strong effect on any shoot or root phenotype in *chk1* mutants, we assessed the effect of a *CHK1* knockdown using RNA interference (RNAi), by targeting regions in the CHASE or HIS domains in wild-type pea plants. RNAi constructs targeting the CHASE domain of CHK1 indeed reduced *CHK1* transcript levels, and not *CHK2, CHK3*, or *CHK4*, compared to the Empty Vector (EV) or to untransformed controls (**Figures 6B, C**; **Supplementary Figure 11**). A small but not significant decrease in *PsCHK1* expression was also observed in wild-type roots transformed with the RNAi construct targeting the HIS domain (**Figure 6B**). At 3 weeks post-inoculation with rhizobium, a significant decrease in the number of developing nodules was observed in plants transformed with RNAi::CHK1_CHASE_ compared to EV transformed roots, and a slight but not significant reduction in mature nodules compared to untransformed roots (**Figures 6A, E**). In addition, CHK1 silencing had a positive effect on the number of infection threads (**Figure 6D**). The comparison of RNAi::CHK1_CHASE_ transformed and untransformed roots at 5 weeks after inoculation showed an even more pronounced suppression of the number of developing and mature nodules (**Figure 6G**). However, no significant effect on rhizobial infections was detected when comparing untransformed and transformed roots (**Figure 6F**). At neither 3 or 5 weeks was there an effect of RNAi::CHK1_CHASE_ transformation on lateral root number (**Figures 6E, G**). There was also no significant effect on nodule or lateral root number when roots were transformed with RNAi::CHK1_HIS_ (**Figure 6E**). These findings indicate that *Ps*CHK1 functions as the primary receptor controlling nodule initiation and development in pea.

## Discussion

In this study, we characterized mutants for the four *CHK* receptor genes in pea. Sequence analysis revealed that mutations in *chk1* and *chk3* occur at conserved amino acid residues; however, only the *chk3* mutation is predicted to have a strong deleterious effect on receptor function, whereas the *chk1* allele likely retains partial activity. This residual function may account for the relatively mild phenotypic alterations observed in *chk1* mutants, especially in root phenotypes where CHK1 orthologues *Mt*CRE1 and *Lj*LHK1 have critical roles. In contrast, the *chk2* and *chk4* alleles result in truncated proteins, which are presumed to produce non-functional receptors (**Figure 1**). Surprisingly, the *chk* quadruple mutant retained the ability to respond to exogenous cytokinin through the upregulation of *RRA* genes, although the spatial pattern of activation of the cytokinin-responsive promoter *TCSn* was altered (**Figure 2**). These findings suggest that at least one receptor, most likely CHK1, retains most functionality and contributes to cytokinin perception in the quadruple mutant. Phenotypic studies of single and higher order mutants revealed specific roles for some CHK receptors, e.g. leaf size, shoot branching and senescence, while for other traits there was clear redundancy between CHK2, CHK3 and CHK4 receptors.

### Cytokinin perception via CHK receptors regulates shoot growth and delays senescence

In pea, different CHK receptors contribute to the promotion of the overall shoot and root growth, as has been previously observed in *Arabidopsis* and rice. Although there was no consistent effect in single and double mutants, the triple and quadruple *chk* mutants displayed significantly smaller shoots and roots, indicating that *CHK* genes redundantly regulate biomass allocation in shoots and roots (**Figures 3**, **5**; **Supplementary Figures 6**-**9**). This is consistent with observations performed in *Arabidopsis ahk* mutants, where triple mutants for *AHK* receptors display a strong reduction in shoot and root size, while single mutants exhibit either no or only mild phenotypes (Higuchi et al., 2004; Riefler et al., 2006). Conversely, overexpression of *Zea mays HK* cytokinin receptor genes, or gain-of-function *ahk* mutations in *Arabidopsis*, result in increased shoot and leaf size, primarily due to an enhanced cell size and number (Wang et al., 2014). Slightly different patterns of *CHK* contributions to regulating stem width and stem length were observed in pea. CHK2, CHK3, and CHK4 promote stem length, but no additive effect was observed in the quadruple mutant. The promotion of stem width requires both CHK3 and CHK4, and due to the additive effect in quadruple mutant possibly also CHK2 (**Figure 3**).

A specific role for CHK3 was found in delaying leaf senescence during plant maturation in pea. Of note, our study monitored leaf senescence in *chk* mutants during plant maturation, extending our understanding of CK role in senescence in mature leaves during vegetative growth. Leaf senescence was indeed significantly accelerated by several weeks in *chk3*, as well as in the quadruple mutant (**Figure 4**). This was not due to changes in flowering time or node (**Supplementary Figure 5**), indicating a likely direct role for CHK3 during natural leaf senescence. This is similar to the previously documented central role of *Os*CHK4 in the delay of rice leaf senescence in response to CK, and differs from the redundant role for *At*HK2 and *At*HK3 in *Arabidopsis* in delay of leaf senescence in response to CK (Kim et al., 2006; Riefler et al., 2006; Chun et al., 2023).

Another clear-cut result obtained is that *Ps*CHK4 acts to promote shoot branching and leaf size. When grown under short day conditions, which enhances branching in the wild-type, the *chk4* single mutant displayed reduced leaf size and number as well as length of branches (**Figure 3**), suggesting that CHK4-mediated cytokinin signaling promotes leaf size and shoot branching. This is consistent with studies in rice reporting that *Os*HK4 promotes tillering (Yao et al., 2023), and with the reduced leaf size previously described in *ahk* mutants and CK-deficient transgenic *Arabidopsis* plants (Werner et al., 2003; Nishimura et al., 2004; Riefler et al., 2006). Surprisingly, the *chk* quadruple pea mutant displayed an increased shoot branch number and length, opposite to the phenotype of the *chk4* single mutant. This suggests that other CHKs might act redundantly to suppress shoot branching. Although CK is known to promote shoot branching based on CK applications and quantification analyses (for review see Beveridge et al., 2023), the role of canonical CK signaling, including type A and type B RRs, in regulating axillary bud release, seems more complex. Indeed, RRAs generally act as negative regulators of cytokinin signaling, whereas RRBs promote cytokinin signaling. Based on this model, mutants lacking type A RRs should display an enhanced branching due to increased cytokinin signaling. Yet, in *Arabidopsis*, hextuple *arrA*-type mutants have a reduced branching (Müller et al., 2015), while *arr1* mutants —defective in a type-B regulator— have an increased branching (Waldie and Leyser, 2018). These findings challenge the classical cytokinin model of shoot branching, and align with the observations gained in the *chk* quadruple mutant, suggesting that cytokinin’s role in shoot branching may involve additional, yet unidentified, regulators.

### *CHK1* promotes nodulation in pea

Our results indicate that CHK1 is the major CK receptor promoting nodule organogenesis in pea. The *chk2*, *chk3*, and *chk4* alleles individually have no measurable effect on nodulation, whereas the *chk1* allele exhibits a mild reduction in nodule number per root dry weight (**Figure 5**). The RNAi-mediated knockdown of *CHK1* independently confirmed that this receptor is the primary CK receptor responsible for the regulation of nodulation in pea. This is consistent with results showing that CHK1 orthologs have a critical role in nodulation across several legumes, although other receptors play redundant roles (Higuchi et al., 2004; Murray et al., 2007; Held et al., 2014; Boivin et al., 2016; Sun et al., 2023). Similar to *Medicago truncatula* (Gonzalez-Rizzo et al., 2006), we found little evidence that CHK1 suppresses the number of rhizobial infection threads, suggesting that in indeterminate nodulators, CK primarily promotes nodule organogenesis. The lack of additive nodulation phenotype in any of the double, triple, and quadruple *chk* mutants in pea indicates no role for the other CHK receptors, in contrast to the somewhat redundant roles played by CHK receptors in regulating *M. truncatula* and *L. japonicus* nodulation (Held et al., 2014; Boivin et al., 2016).

We did not observe any change in lateral root number in single or higher order *chk* mutants of pea, including the quadruple or any change in the number of lateral roots in *Ps*CHK1 RNAi transformed roots. However, a knock-out *chk1* mutant allele in pea would be useful to more fully characterize the role of CK signaling via CHKs on the root system architecture of legumes, given that the CHK1 orthologs *Mt*CRE1, *Gm*CRE1, and *Lj*LHK1 have been shown to influence lateral root number in different ways. Indeed, *Mtcre1* mutants and *MtCRE1* RNAi lines exhibit an increased number of lateral roots, and CK application suppresses lateral root formation in this species (Gonzalez-Rizzo et al., 2006; Laffont et al., 2015), suggesting that CK signaling suppresses lateral root formation. However, in Lotus, *Lj*LHK1 and *Lj*HK3 mutants display a reduced lateral root number (Held et al., 2014), and similarly, CRISPR *GmCRE1a/b/c/d* lines in soybean also exhibit a reduced lateral root number (Sun et al., 2023).

In conclusion, our findings indicate that individual CHK receptors contribute to distinct developmental functions across organs: *Ps*CHK4 likely promotes shoot branching and leaf size, *Ps*CHK2 and *Ps*CHK4 promote shoot elongation, *Ps*CHK3 delays leaf senescence, and *Ps*CHK1 predominantly regulates nodulation. The pea *chk* mutants generated in this study therefore represent a valuable resource for dissecting cytokinin function across these various developmental contexts. Combined with targeted gene-silencing approaches, such as RNAi, these mutants revealed new insights into cytokinin signaling networks in pea. Moreover, they offer an excellent genetic framework for further exploring cytokinin’s role in branching, reinforcing pea’s position as a model system for studying shoot architecture and hormonal interactions.

## Data Availability

The original contributions for this study are included in the article or Supplementary figures and tables. The raw data supporting the conclusions of this article will be made available by the authors, without undue reservation.

## Supplementary material

Supplementary Figure 1 Representative RFLP genotyping of CHK1–CHK4 loci in Pisum sativum segregating populations. RFLP analysis confirming the presence of mutations in CHK1, CHK2, CHK3, and CHK4. Representative F2 individuals from segregating populations are shown for each locus. Bands corresponding to wild-type (WT), heterozygous (Het), and homozygous mutant (Mut) genotypes are indicated. Homozygous mutants were selected through successive crosses to generate higher-order (quadruple) mutants. This panel illustrates the expected banding patterns for each genotype, documenting the stepwise genotyping strategy used to confirm homozygosity in the quadruple mutants.

Supplementary Figure 2 Phylogenetic relationships and cytokinin-responsive expression of pea response regulator type A (RRA) genes. **(A)** Phylogenetic tree of RRA genes from *Pisum sativum*, *Medicago truncatula*, and *Arabidopsis thaliana*. Sequences of *M. truncatula* and *A. thaliana* RRA genes were used to identify *P. sativum* RRA orthologs. The evolutionary history was inferred using the Maximum Likelihood method with the Tamura–Nei model in MEGA11. The bootstrap consensus tree was generated from 1000 replicates, with bootstrap values (≥50%) shown next to the branches. Initial trees for the heuristic search were obtained automatically using the Neighbor-Join and BioNJ algorithms, selecting the topology with the highest log-likelihood value. **(B)** Relative expression levels of *RRA* genes normalized to *TFII* expression in wild-type and *chk* quadruple mutant roots and leaves, 60 min after treatment with 6-benzylaminopurine (6-BAP). Values represent means ± standard error (n = 5). Different letters indicate statistically significant differences (one-way ANOVA followed by Tukey’s *post hoc* test).

Supplementary Figure 3 CK response patterns wild-type (Cameor) and *chk* quadruple mutant pea plants transformed with *TCSn::GUS*. Roots were stained for β-glucuronidase (GUS) activity, blue color indicates areas of high cytokinin response. **(A)** Bright field image of developing nodules, arrows indicates nodule primordial **(B)** Bright field image of mature nodules **(C)** Representative images of whole roots (scale bar is 5cm).

Supplementary Figure 4 Shoot phenotype in *chk2* single and wild type. **(A)** Representative shoot phenotypes of wild-type (WT), single *chk2*. Plants were grown for 8 weeks after planting (2 plants per pot). **(B, C)** Quantitative analysis of shoot traits for WT and *chk2* mutant. n = 6. **(D)** Branching and flowering node, leaf area and shood width of WT and *chk2* mutant plants. Bars represent means ± standard error. Different letters indicate statistically significant differences within a parameter (p < 0.05), as determined by t-test.

Supplementary Figure 5 Flowering node in *chk* single and quadruple mutants. Quantitative analysis of first flowering node in wild-type (WT), single *chk* mutants, and the *chk1chk2chk3chk4* quadruple mutant (ANOVA, p = 0.14). WT and single mutants: *n* = 6; quadruple mutant: *n* = 10. Bars represent means ± standard error. Different letters indicate statistically significant differences within a parameter (*p* < 0.05), as determined by one-way ANOVA followed by Tukey’s *post hoc* test.

Supplementary Figure 6 Shoot phenotype of *chk1*, *chk2*, *chk3*, *chk4* single mutants and the *chk1chk2chk3chk4* quadruple mutant in nodulation experiments. **(A)** Representative images of plants from each genotype in nodulation experiment. **(B)** Quantitative analysis of shoot and root traits in nodulation experiment: shoot dry weight (Shoot DW) (ANOVA, p = 2e–16; n = 12), root dry weight (Root DW) (ANOVA, p = 0.00021; n = 3) and number of secondary roots (2ry roots) (ANOVA, p = 0.0151; n = 3). Bars represent means ± standard error. Different letters indicate statistically significant differences within a parameter (*p* < 0.05), as determined by one-way ANOVA followed by Tukey’s *post hoc* test.

Supplementary Figure 7 Nodulation phenotype of double mutants *chk1chk2*, *chk1chk3*, *chk1chk4*, and *chk3chk4*. **(A)** Representative images of plants from each genotype. **(B)** Quantitative analysis of shoot and root traits: shoot dry weight (Shoot DW) (ANOVA, p = 0.00022; n = 10), root dry weight (Root DW) (ANOVA, p = 0.0424; n = 5), total number of nodules per plant (#Nodules) (ANOVA, p = 0.0437; n = 5), and number of secondary roots (2ry roots) (ANOVA, p = 0.0205; n = 5). **(C)** Nodule-related and branching traits: nodule dry weight (Nodule DW) (ANOVA, p = 0.516; n = 5), number of nodules per root dry weight (Nodules/RDW) (ANOVA, p = 0.449; n = 5, and individual nodule weight (ANOVA, p = 0.00227; n = 5). **(D, E)** Quantification of nodulation structures per centimeter of roots of wild type, *chk1chk3 and chk1chk4*: **(D)** infection threads (ANOVA, p = 0.945; n = 6), and **(E)** developing (ANOVA, p = 0.0441; n = 6) and mature nodules (ANOVA, p = 0.311; n = 6), counted under a microscope using GFP-tagged rhizobia. Bars represent means ± standard error. Different letters indicate statistically significant differences within a parameter (*p* < 0.05), as determined by one-way ANOVA followed by Tukey’s *post hoc* test or Kruskal–Wallis test with Bonferroni correction.

Supplementary Figure 8 Nodulation phenotype of the triple mutant *chk1chk3chk4*. **(A)** Quantitative analysis of shoot and root traits: shoot dry weight (Shoot DW) (t-test, p = 0.0070; n = 12), root dry weight (Root DW) (t-test, p = 0.434; n = 3), total number of nodules per plant (#Nodules) (t-test, p = 0.908; n = 3), and number of secondary roots (2ry roots) (t-test, p = 0.734; n = 3). **(B)** Nodule-related and branching traits: nodule dry weight (Total NDW) (t-test, p = 0.571; n = 3), number of nodules per root dry weight (Nodules/RDW) (t-test, p = 0.0462; n = 3), and individual nodule weight (t-test, p = 0.43; n = 3). **(C)** Quantification of nodulation structures per centimeter of root: total infection threads (TIT) (t-test, p = 0.1303; n = 12), developing nodules (t-test, p = 0.455; n = 12), and mature nodules (t-test, p = 0.9618; n = 12), counted under the microscope using GFP-tagged rhizobia. **(D)** Representative images of typical plants for each genotype. Bars represent means ± standard error. Different letters indicate statistically significant differences (p < 0.05) within a parameter.

Supplementary Figure 9 Nodulation phenotype of the triple mutants *chk1chk2chk3* and *chk1chk2chk4*. **(A)** Quantitative analysis of shoot and root traits: shoot dry weight (Shoot DW) (ANOVA, p = 0.00078; n = 12), root dry weight (Root DW) (ANOVA, p = 0.159; n = 3–4), total number of nodules per plant (#Nodules) (ANOVA, p = 0.027; n = 3–4), and number of secondary roots (2ry roots) (ANOVA, p = 0.106; n = 3–4). **(B)** Nodule-related and branching traits: nodule dry weight (Nodule DW) (ANOVA, p = 0.585; n = 3–4), number of nodules per root dry weight (Nodules/RDW) (ANOVA, p = 0.0535; n = 3–4), and individual nodule weight (ANOVA, p = 0.347; n = 3–4). **(C, D)** Quantification of nodulation structures per centimeter of root for **(C)** infection threads (ANOVA, p = 0.19; n = 8) and **(D)** developing (ANOVA, p = 0.0205; n = 8) and mature nodules (ANOVA, p = 0.078; n = 8), counted under the microscope using GFP-tagged rhizobia. **(E)** Representative images of typical plants for each genotype. Values represent means ± standard error. Grouping letters were obtained using one-way ANOVA with Tukey’s *post hoc* test or Kruskal–Wallis test with Bonferroni correction. Values with different letters are significantly different (p < 0.05) within a parameter.

Supplementary Figure 10 Leaf senescence in *chk* single and quadruple mutants. Leaf color over time on leaflet 4 of wild-type (WT), single *chk1*, *chk2*, *chk3*, *chk4*, and quadruple *chk1chk2chk3chk4* mutants. The same leaf was imaged every week from 1–2 weeks after planting.

Supplementary Figure 11 Relative expression of *CHK2*, *CHK3*, and *CHK4* genes in the *CHK1* RNAi knockdown assay. Relative expression of *CHK2*, *CHK3*, and *CHK4* in roots transformed with empty vector (EV), *RNAi::CHK1_CHASE_*, or *RNAi::CHK1_HIS_* constructs (*n* = 8). Expression values were normalized to the geometric mean of reference genes *Actin7* and *TFIIa*. Box plots show the median, interquartile range, and individual data points. Values with the same letters are not significantly different.
