# Supplementary material for: The cytokinin histidine kinase receptors regulate nodulation, shoot and root development in Pisum sativum
Source: Front Plant Sci. 2026 Mar 16;17:1750990. doi: 10.3389/fpls.2026.1750990 (PMC13034853; doi:10.3389/fpls.2026.1750990)
Supplement: Supplementary Figure 1 — Representative RFLP genotyping of CHK1–CHK4 loci in Pisum sativum segregating populations. RFLP analysis confirming the presence of mutations in CHK1, CHK2, CHK3, and CHK4. Representative F2 individuals from segregating populations are shown for each locus. Bands corresponding to wild-type (WT), heterozygous (Het), and homozygous mutant (Mut) genotypes are indicated. Homozygous mutants were selected through successive crosses to generate higher-order (quadruple) mutants. This panel illustrates the expected banding patterns for each genotype, documenting the stepwise genotyping strategy used to confirm homozygosity in the quadruple mutants. [file DataSheet1.pdf]

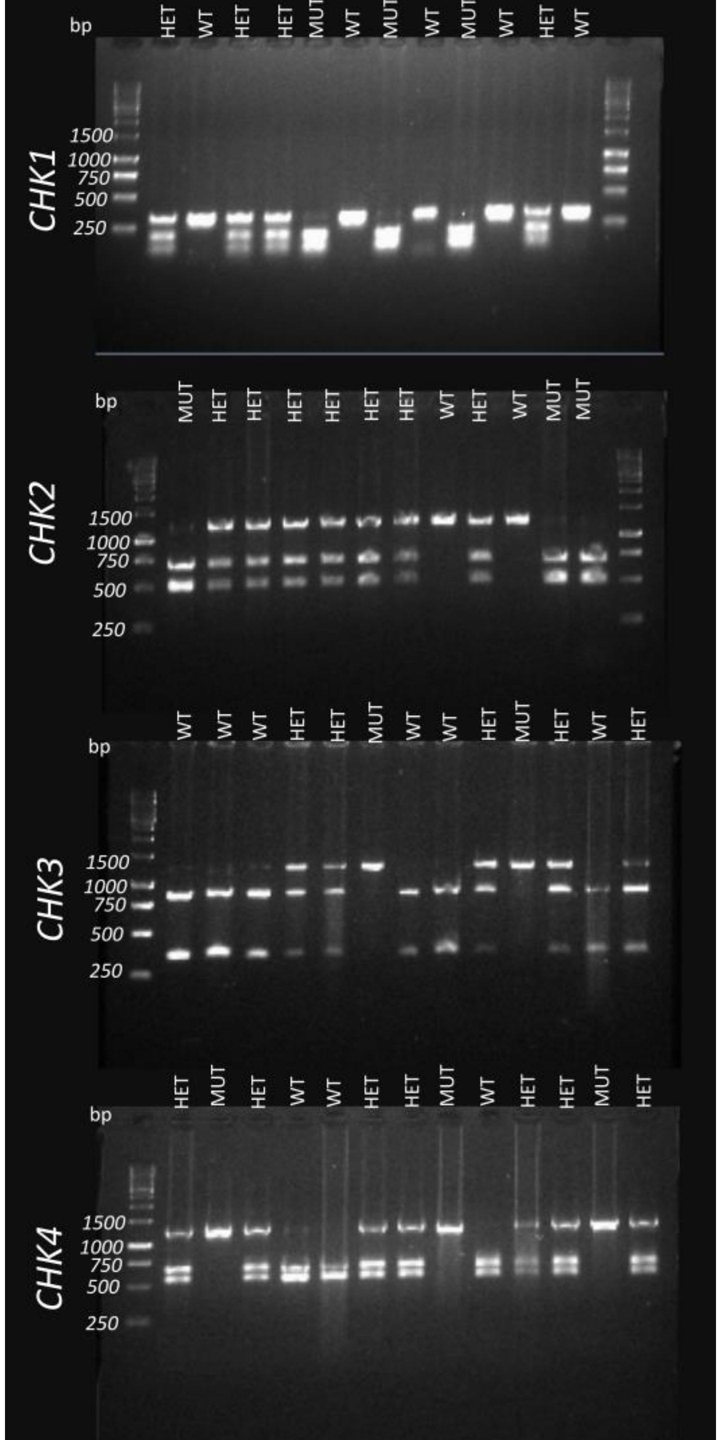

Figure S1. Representative RFLP genotyping of CHK1–CHK4 loci in Pisum sativum segregating populations

RFLP analysis confirming the presence of mutations in CHK1, CHK2, CHK3, and CHK4. Representative F2 individuals from segregating populations are shown for each locus. Bands corresponding to wild-type (WT), heterozygous (Het), and homozygous mutant (Mut) genotypes are indicated. Homozygous mutants were selected through successive crosses to generate higher-order (quadruple) mutants. This panel illustrates the expected banding patterns for each genotype, documenting the stepwise genotyping strategy used to confirm homozygosity in the quadruple mutants.

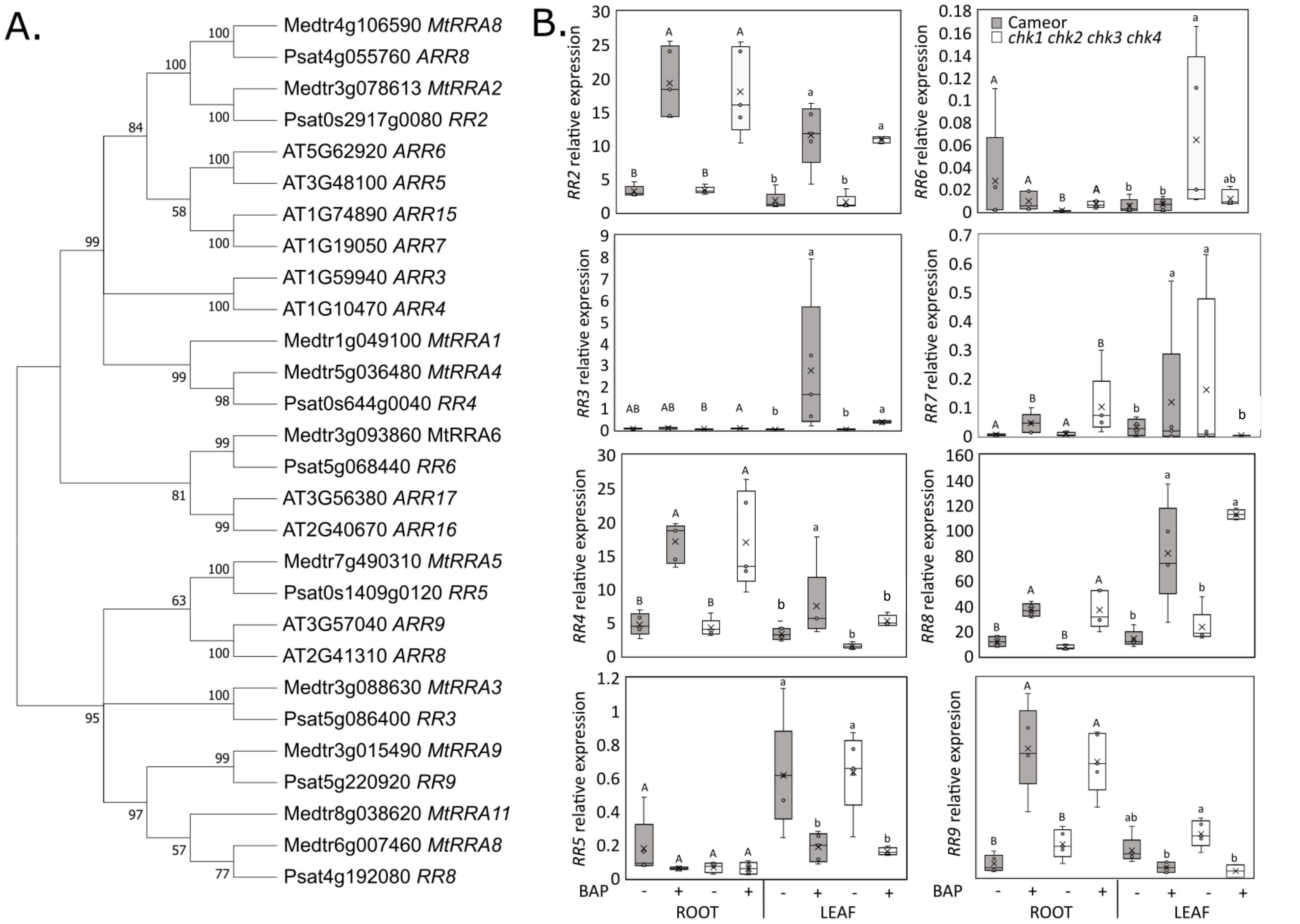

Figure S2. Phylogenetic relationships and cytokinin-responsive expression of pea response regulator type A (*RRA*) genes. **(A)** Phylogenetic tree of RRA genes from *Pisum sativum*, *Medicago truncatula*, and *Arabidopsis thaliana*. Sequences of *M. truncatula* and *A. thaliana* RRA genes were used to identify *P. sativum* RRA orthologs. The evolutionary history was inferred using the Maximum Likelihood method with the Tamura–Nei model in MEGA11. The bootstrap consensus tree was generated from 1000 replicates, with bootstrap values (≥50%) shown next to the branches. Initial trees for the heuristic search were obtained automatically using the Neighbor-Join and BioNJ algorithms, selecting the topology with the highest log-likelihood value. **(B)** Relative expression levels of *RRA* genes normalized to *TFII* expression in wild-type and *chk* quadruple mutant roots and leaves, 60 min after treatment with 6-benzylaminopurine (6-BAP). Values represent means ± standard error (n = 5). Different letters indicate statistically significant differences (one-way ANOVA followed by Tukey's post hoc test).

Figure S3. CK response patterns wild-type (Cameor) and *chk* quadruple mutant pea plants transformed with *TCSn::GUS*. Roots were stained for β-glucuronidase (GUS) activity, blue colour indicates areas of high cytokinin response.

(A) Bright field image of developing nodules, arrows indicates nodule primordia

(B) Bright field image of mature nodules

(C) Representative images of whole roots (scale bar is 5cm)

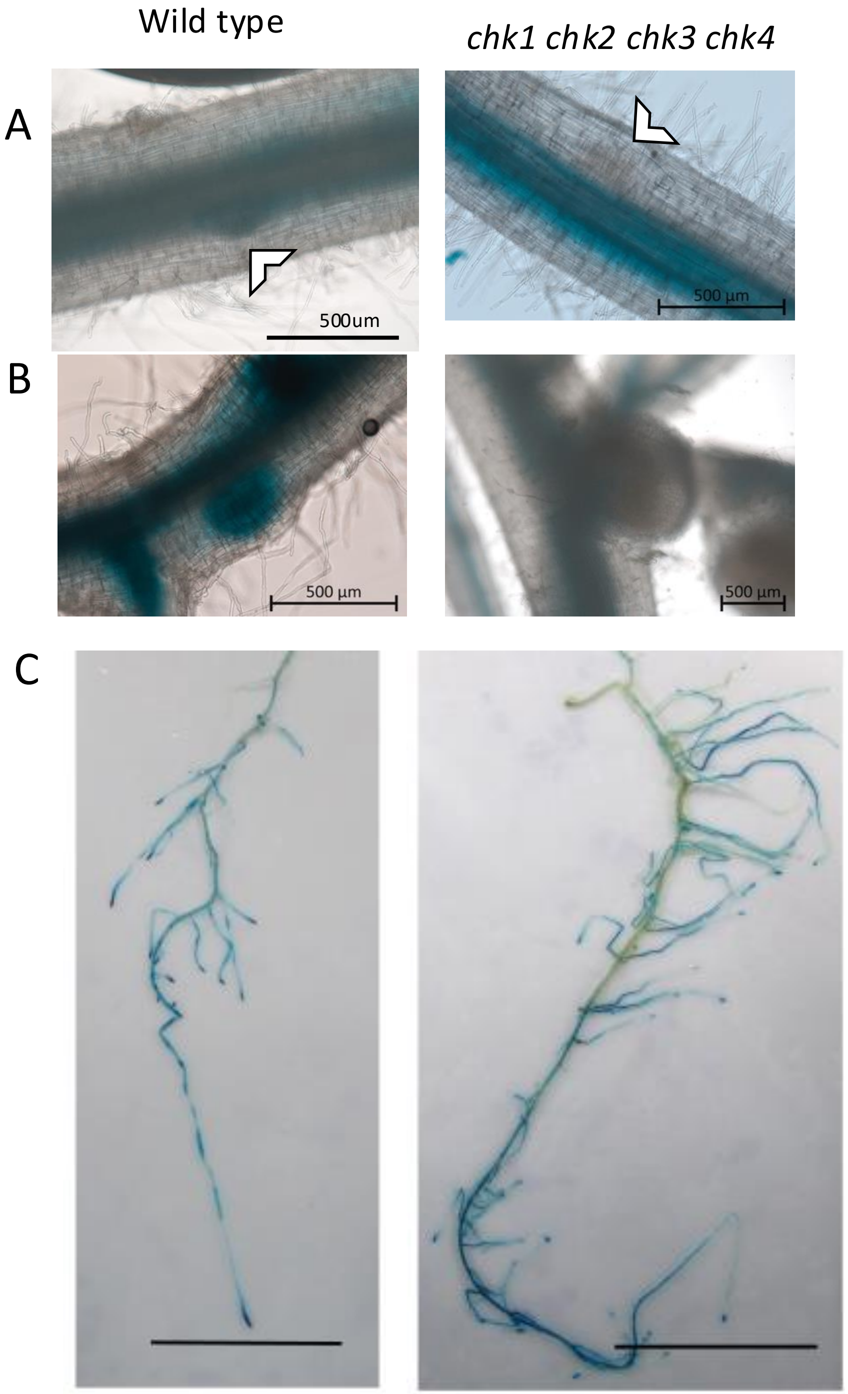

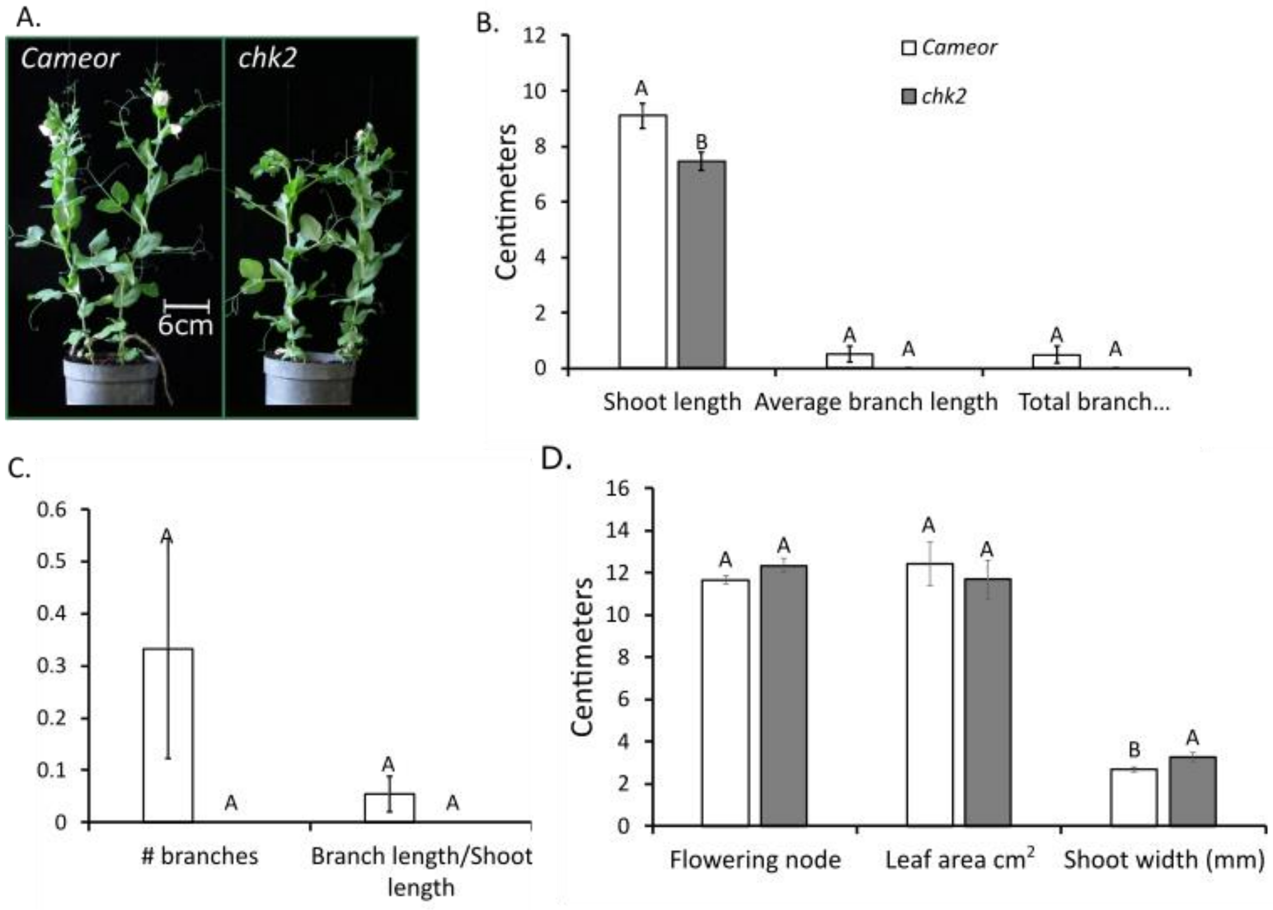

Figure S4. Shoot phenotype in *chk2* single and wild type

(A) Representative shoot phenotypes of wild-type (WT), single *chk2*. Plants were grown for 8 weeks after planting (2 plants per pot).

(B-C) Quantitative analysis of shoot traits for WT and *chk2* mutant. n = 6.

(D) Branching and flowering node, leaf area and shood width of WT and *chk2* mutant plants. Bars represent means ± standard error. Different letters indicate statistically significant differences within a parameter ($p < 0.05$), as determined by t-test.

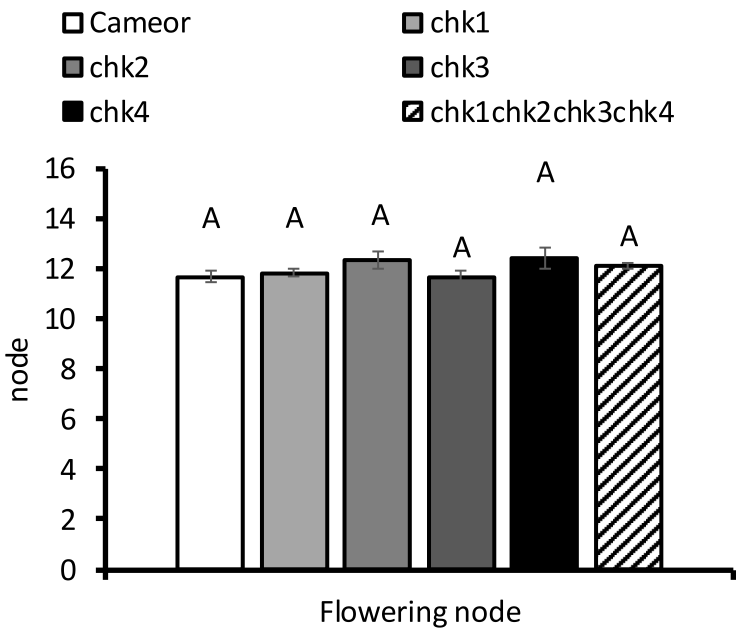

Figure S5. Flowering node in *chk* single and quadruple mutants.
Quantitative analysis of first flowering node in wild-type (WT), single *chk* mutants, and the *chk1chk2chk3chk4* quadruple mutant (ANOVA, p = 0.14). WT and single mutants: n = 6; quadruple mutant: n = 10. Bars represent means ± standard error. Different letters indicate statistically significant differences within a parameter ($p < 0.05$), as determined by one-way ANOVA followed by Tukey's post hoc test.

A.

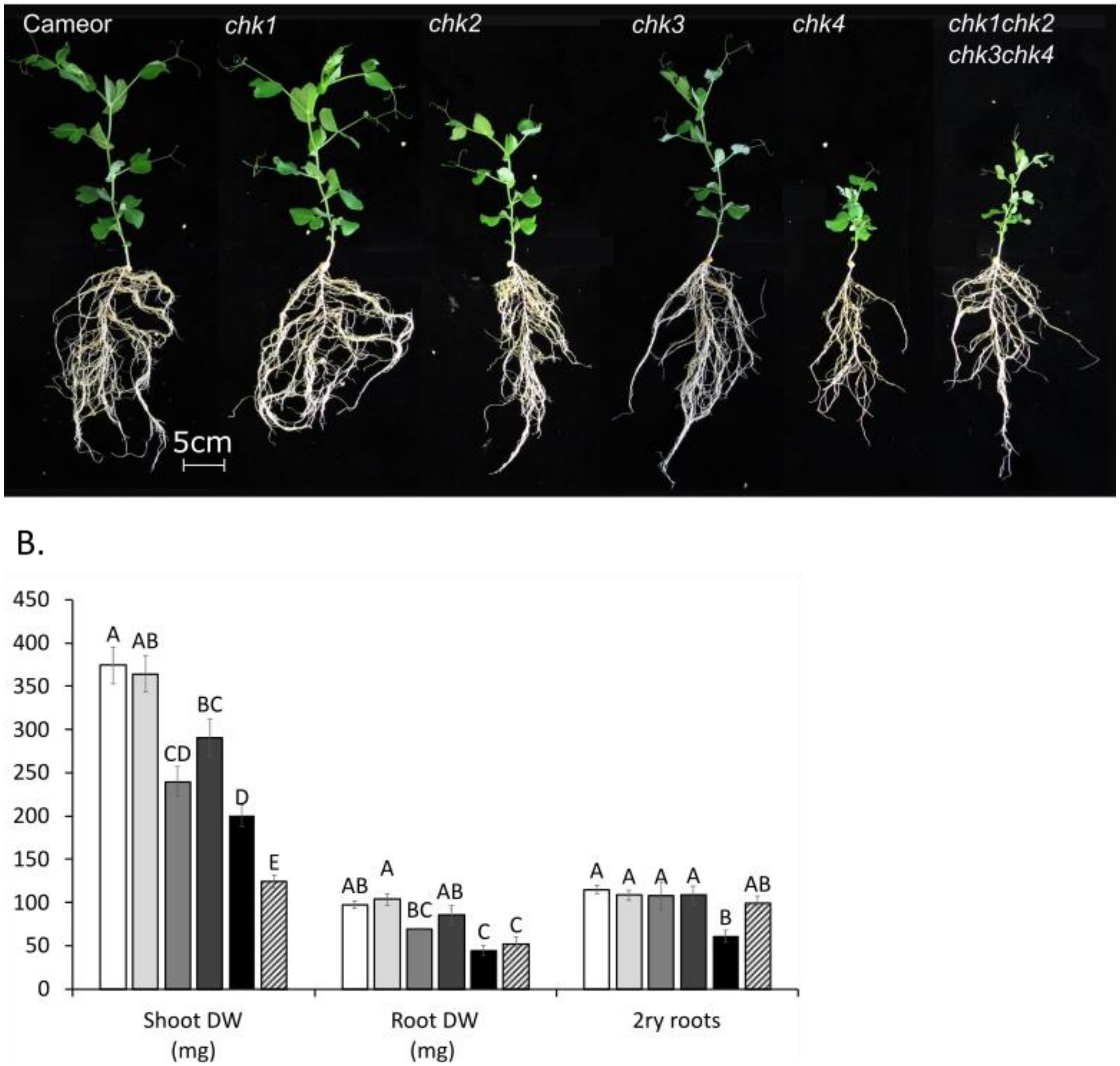

Figure S6. Shoot phenotype of *chk1*, *chk2*, *chk3*, *chk4* single mutants and the *chk1chk2chk3chk4* quadruple mutant in nodulation experiment.

(A) Representative images of plants from each genotype in nodulation experiment.

(B) Quantitative analysis of shoot and root traits in nodulation experiment: shoot dry weight (Shoot DW) (ANOVA, p = 2e–16; n = 12), root dry weight (Root DW) (ANOVA, p = 0.00021; n = 3) and number of secondary roots (2ry roots) (ANOVA, p = 0.0151; n = 3). Bars represent means ± standard error. Different letters indicate statistically significant differences within a parameter ($p < 0.05$), as determined by one-way ANOVA followed by Tukey's post hoc test.

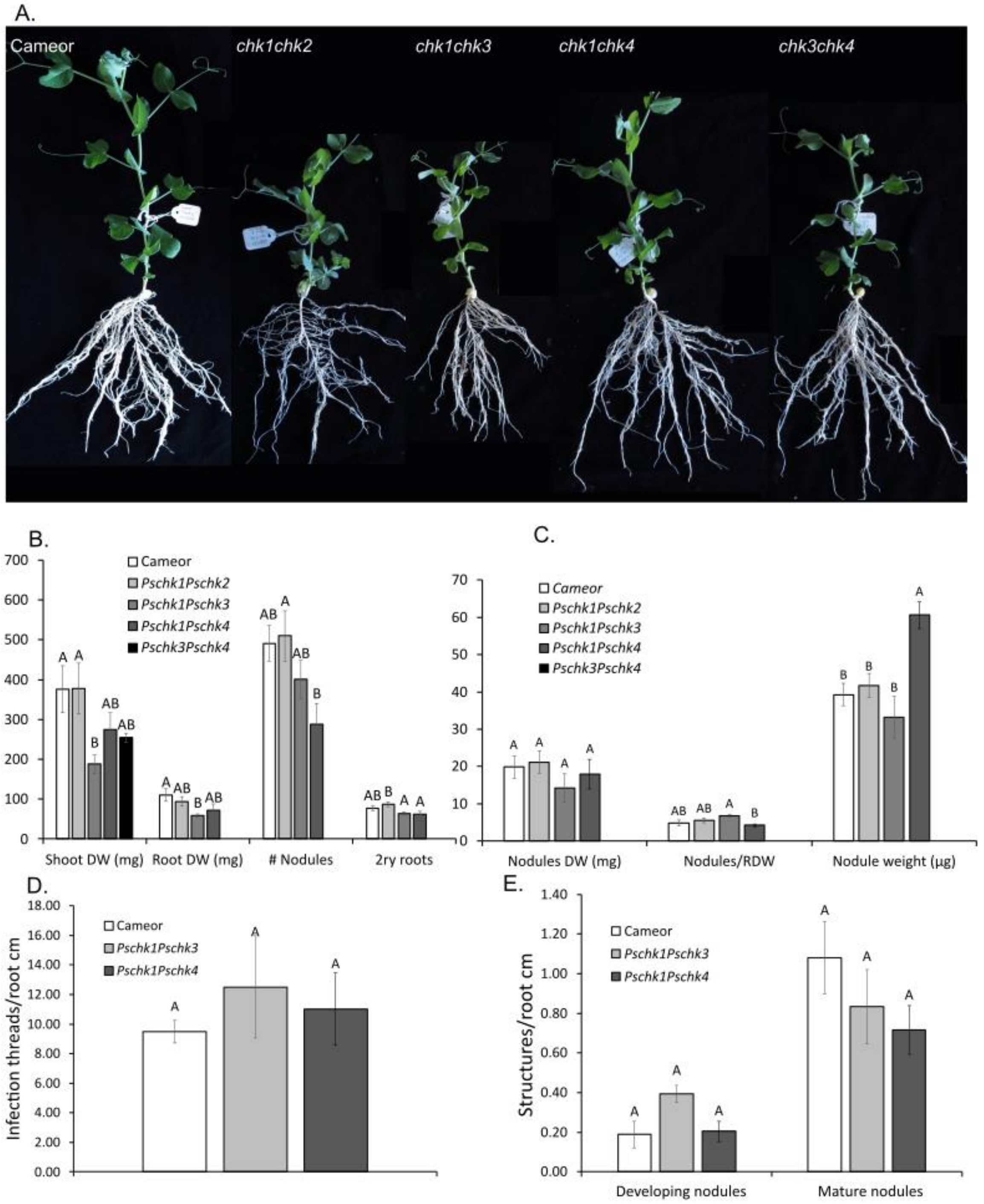

Figure S7. Nodulation phenotype of double mutants *chk1chk2*, *chk1chk3*, *chk1chk4*, and *chk3chk4*.

(A) Representative images of plants from each genotype.

(B) Quantitative analysis of shoot and root traits: shoot dry weight (Shoot DW) (ANOVA, $p = 0.00022$; $n = 10$), root dry weight (Root DW) (ANOVA, $p = 0.0424$; $n = 5$), total number of nodules per plant (#Nodules) (ANOVA, $p = 0.0437$; $n = 5$), and number of secondary roots (2ry roots) (ANOVA, $p = 0.0205$; $n = 5$).

(C) Nodule-related and branching traits: nodule dry weight (Nodule DW) (ANOVA, $p = 0.516$; $n = 5$), number of nodules per root dry weight (Nodules/RDW) (ANOVA, $p = 0.449$; $n = 5$, and individual nodule weight (ANOVA, $p = 0.00227$; $n = 5$).

(D–E) Quantification of nodulation structures per centimetre of roots of wild type, *chk1chk3 and chk1chk4* : (D) infection threads (ANOVA, $p = 0.945$; $n = 6$), and (E) developing (ANOVA, $p = 0.0441$; $n = 6$) and mature nodules (ANOVA, $p = 0.311$; $n = 6$), counted under a microscope using GFP-tagged rhizobia.

Bars represent means ± standard error. Different letters indicate statistically significant differences within a parameter ($p < 0.05$), as determined by one-way ANOVA followed by Tukey's post hoc test or Kruskal–Wallis test with Bonferroni correction.

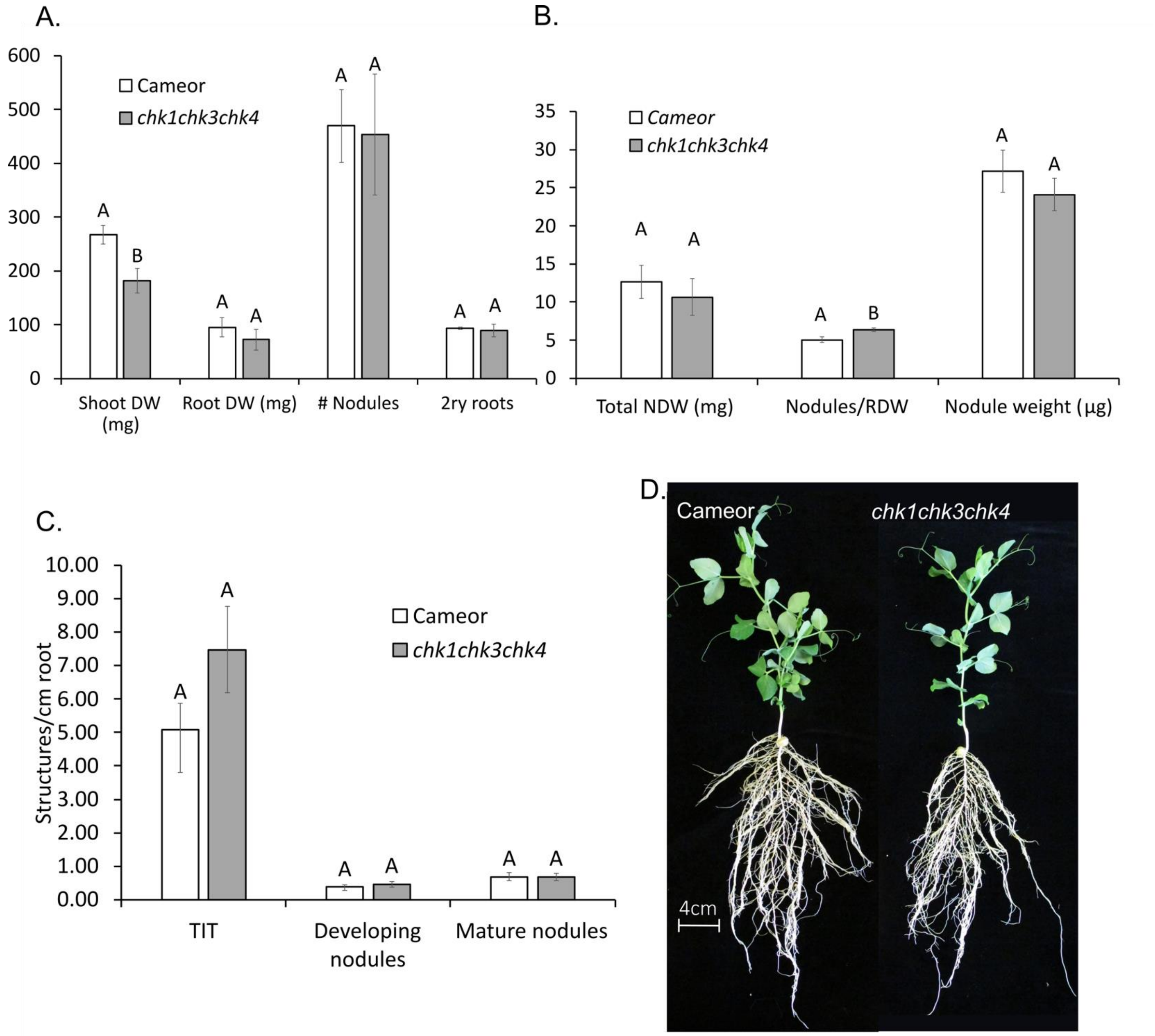

Figure S8. Nodulation phenotype of the triple mutant *chk1chk3chk4*.
(A) Quantitative analysis of shoot and root traits: shoot dry weight (Shoot DW) (t-test, $p = 0.0070$; $n = 12$), root dry weight (Root DW) (t-test, $p = 0.434$; $n = 3$), total number of nodules per plant (#Nodules) (t-test, $p = 0.908$; $n = 3$), and number of secondary roots (2ry roots) (t-test, $p = 0.734$; $n = 3$).
(B) Nodule-related and branching traits: nodule dry weight (Total NDW) (t-test, $p = 0.571$; $n = 3$), number of nodules per root dry weight (Nodules/RDW) (t-test, $p = 0.0462$; $n = 3$), and individual nodule weight (t-test, $p = 0.43$; $n = 3$).
(C) Quantification of nodulation structures per centimetre of root: total infection threads (TIT) (t-test, $p = 0.1303$; $n = 12$), developing nodules (t-test, $p = 0.455$; $n = 12$), and mature nodules (t-test, $p = 0.9618$; $n = 12$), counted under the microscope using GFP-tagged rhizobia.
(D) Representative images of typical plants for each genotype.
Bars represent means ± standard error. Different letters indicate statistically significant differences ($p < 0.05$) within a parameter.

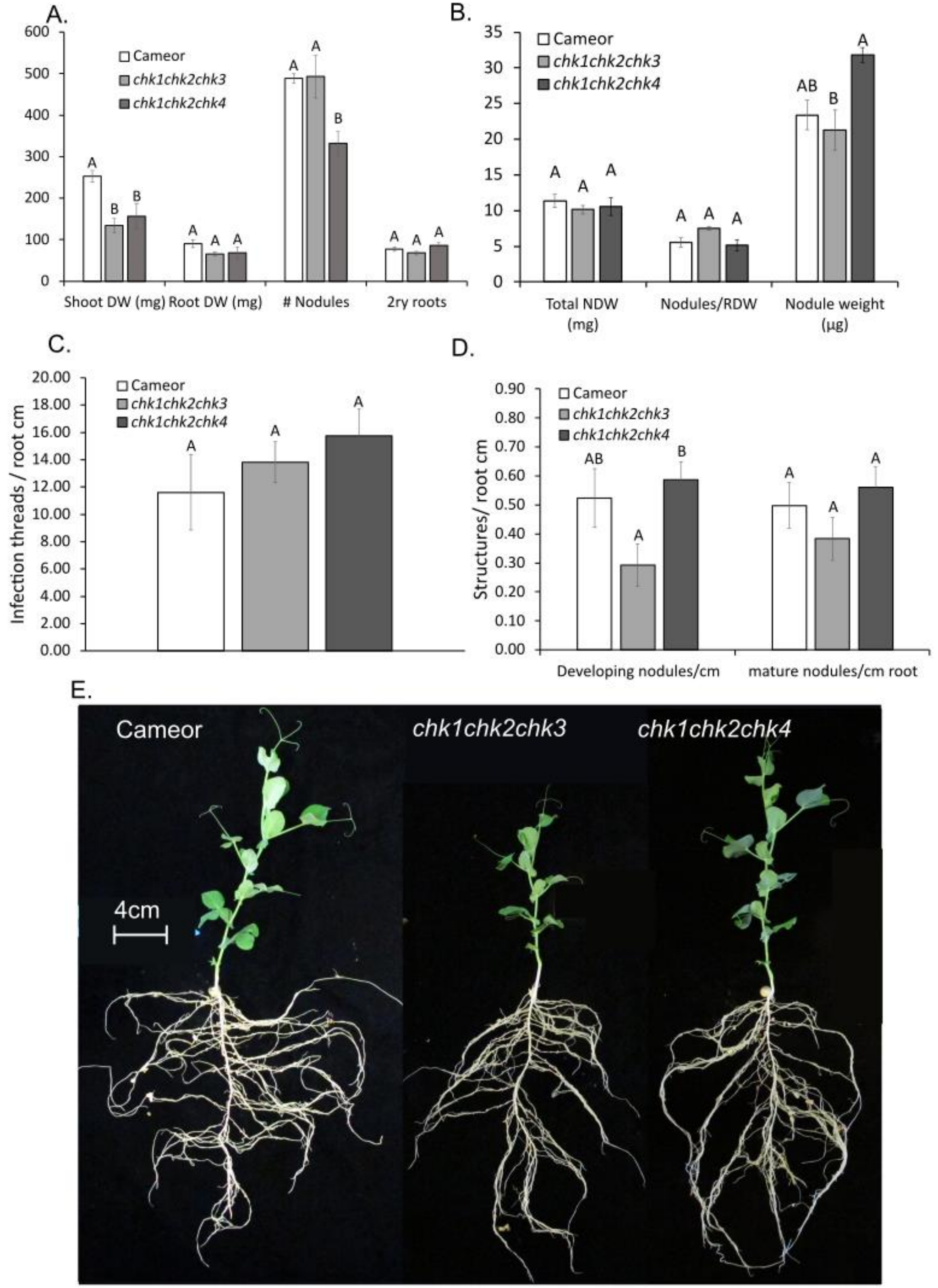

Figure S9. Nodulation phenotype of the triple mutants *chk1chk2chk3* and *chk1chk2chk4*.
(A) Quantitative analysis of shoot and root traits: shoot dry weight (Shoot DW) (ANOVA, $p = 0.00078$; $n = 12$), root dry weight (Root DW) (ANOVA, $p = 0.159$; $n = 3–4$), total number of nodules per plant (#Nodules) (ANOVA, $p = 0.027$; $n = 3–4$), and number of secondary roots (2ry roots) (ANOVA, $p = 0.106$; $n = 3–4$).
(B) Nodule-related and branching traits: nodule dry weight (Nodule DW) (ANOVA, $p = 0.585$; $n = 3–4$), number of nodules per root dry weight (Nodules/RDW) (ANOVA, $p = 0.0535$; $n = 3–4$), and individual nodule weight (ANOVA, $p = 0.347$; $n = 3–4$).
(C–D) Quantification of nodulation structures per centimetre of root for (C) infection threads (ANOVA, $p = 0.19$; $n = 8$) and (D) developing (ANOVA, $p = 0.0205$; $n = 8$) and mature nodules (ANOVA, $p = 0.078$; $n = 8$), counted under the microscope using GFP-tagged rhizobia.
(E) Representative images of typical plants for each genotype.
Values represent means ± standard error. Grouping letters were obtained using one-way ANOVA with Tukey's post hoc test or Kruskal–Wallis test with Bonferroni correction. Values with different letters are significantly different ($p < 0.05$) within a parameter.

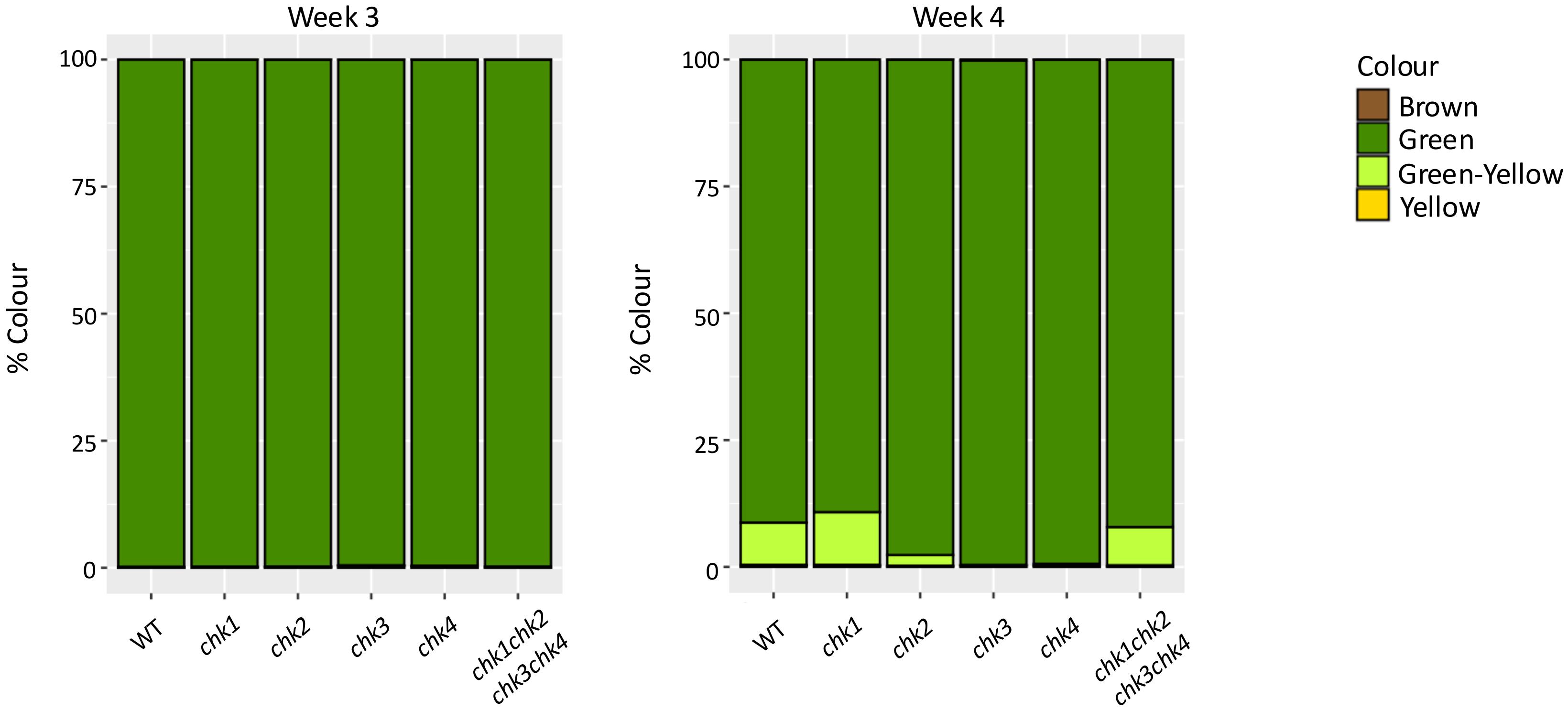

Fig S10. **Leaf senescence in *chk* single and quadruple mutants.**
Leaf colour over time on leaflet 4 of wild-type (WT), single *chk1*, *chk2*, *chk3*, *chk4*, and quadruple *chk1chk2chk3chk4* mutants. The same leaf was imaged every week from 1-2 weeks after planting.

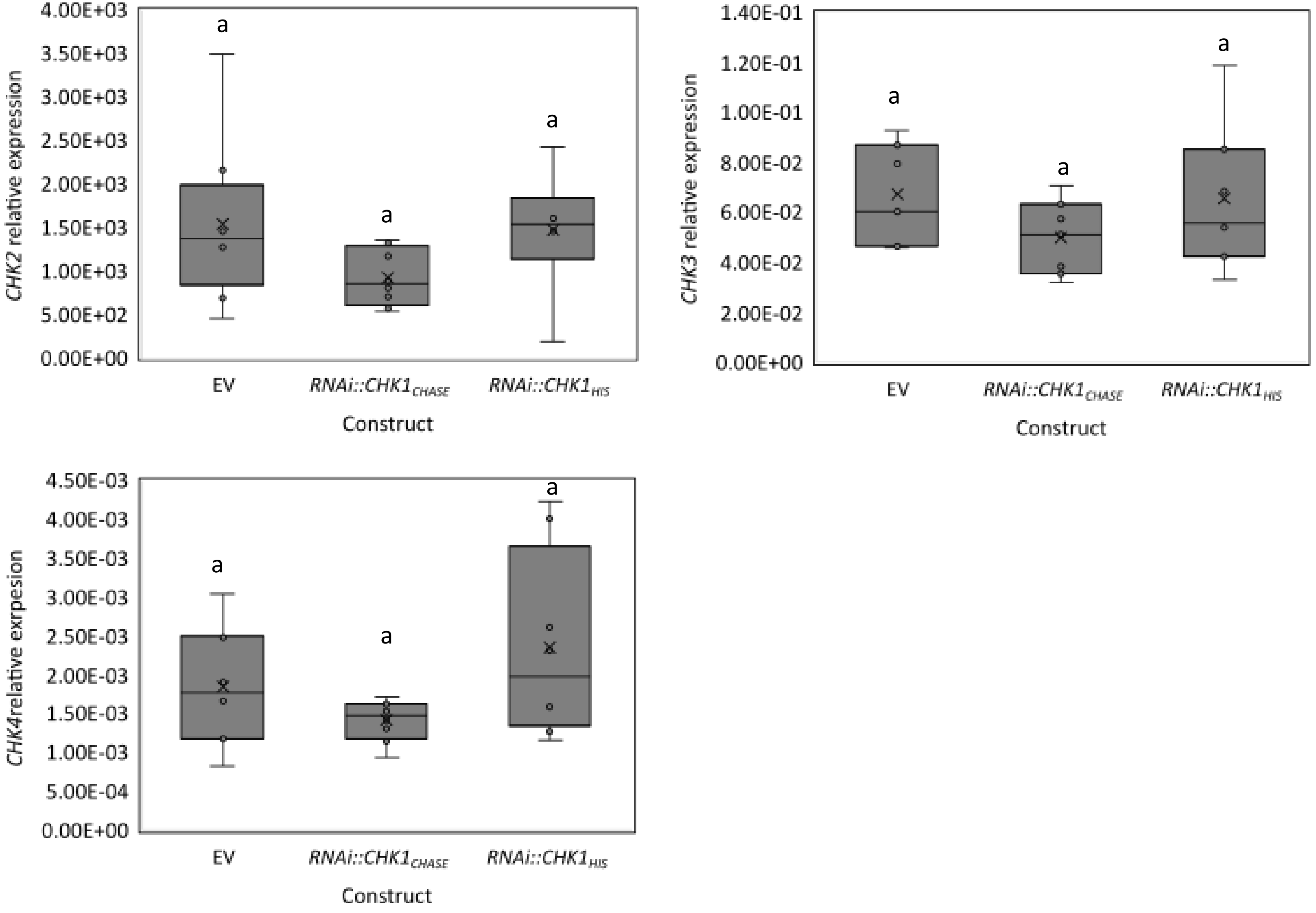

Figure S11. Relative expression of *CHK2*, *CHK3*, and *CHK4* genes in the *CHK1* RNAi knockdown assay.

Relative expression of *CHK2*, *CHK3*, and *CHK4* in roots transformed with empty vector (EV),*RNAi::CHK1*$_{CHASE}$, or *RNAi::CHK1*$_{HIS}$ constructs (n = 8). Expression values were normalized to the geometric mean of reference genes *Actin7* and *TFIIa*. Box plots show the median, interquartile range, and individual data points. Values with the same letters are not significantly different.

Table S1. Primers and genotyping details for *chk* alleles.

Primer sequences, expected amplicon sizes, and corresponding restriction endonucleases used to distinguish wild-type and mutant alleles of the four *CHK* receptor genes are shown. The expected fragment patterns after enzymatic digestion are indicated for both wild-type and mutant genotypes.

Gene	Primers	Fragment size	Enzyme	Expected result
CHK1	PsCHK1/CRE1F 5'CTGATGAGCGCAACAACTGG3'<br>PsCHK1/CRE1R 5'AAAAGTGCTTCCAACCTGCG3'	317bp	Sau3AI	Wild type No cut: 317bp<br>Mutant cut: 121bp+196bp
CHK2	PsCHK2F 5'CTGATTCTATAACTGTATTGCAGT3'<br>PsCHK2R 5'TTGTGGCAACAAGTTAGAGTT3'	1203bp	AflII	Wild type No cut: 1203bp<br>Mutant cut: 698bp+505bp
CHK3	PsCHK3F 5'GTGAAAACTGAATTATGCGTTCG3'<br>PsCHK3R 5'TATCAAGACGTTAGAAGAAAGAATTCAAGC3'	1225bp	BsrI	Wild type cut: 364bp+865bp<br>Mutant no cut: 1225bp
CHK4	PsCHK4F 5'GGACATGTTTTTGTCTCGATTCATC3'<br>PsCHK4R 5'TACCCGTCCATTTCTGGCATTTGG3'	1211bp	PstI	Wild type cut: 557+664<br>Mutant No cut: 1211bp

Table S2. List of primers used for quantitative PCR (qPCR) analysis and TILLING.

Gene	Primer name	Sequence 5'-3'
PsTFIIa	PsTFIIa-F	CGGTGGAAATGCTGATGTTA
	PsTFIIa-R	GCTCCCTCCACATACCTCAA
PsActin 7	PsActinF	ATTGCATTGCACTTTGTGTAGCC
	PsActinR	CCATCTTTTACAAAAACGTGAAA
GUS	qGUSF	CGACTGGGCAGATGAACATG
	qGUSR	GTTCAGGCACAGCACATCAA
PsRR2	qPsRR2F	CACTTCTTGCAAAGTGACCGTTG
	qPsRR2	CCTCCAAGCAACTATCGATTCGG
PsRR4	qPsRR4F	CAGCTTGTAAAGTTACTGCTG
	qPsRR4R	TCCAAACATCTGTCTATGCGT
PsRR8	qPsRR8F	CATGCAAAGTTACTGTTGTGG
	qPsRR8R	TCCTTCCTCAAGGCATCTATC
PsCHK1	qPsCHK1F	GATATGTAGGTATCACCAG
	qPsCHK1R	TGAGAGACAGTAGCTAGAAA
PsCHK2	qPsCHK2F	GCCATTGATCAGAGGACTTTTGC
	qPsCHK2R	GGAGATGGCTCCAATGCTTCT
PsCHK3	qPsCHK3F	TTCAGGAAAGGAAGACCGTG
	qPsCHK3R	TTTCAAACACCCCGCCAAGA
PsCHK4	qPsCHK4F	ATGTCAGGGAAGGAAGATCG
	qPsCHK4R	GATGCGCCCAAATACCCGA
PsCHK1	PsCRE1-F0	ACCCATGCTTGTCTCAATTTCC
	PsCRE1-R0	CGTATATTCCGCAAAGGTTTCCT
	PsCRE1-F2	TTCCCTACACGACGCTCTTCCGATCTAAAAGTGTCTCAGATTTCGG
	PsCRE1-R1	AGTTCAGACGTGTGCTCTTCCGATCTACTTTCAAGCACAAACCTGG
PsCHK3	PsCHK3-F0	CACTGCTGCTTCATTTATTGAGC
	PsCHK3-R0	TATTCACCTAAGGGCTAGGC
	PsCHK3-F2	TTCCCTACACGACGCTCTTCCGATCTGTTGTGGATGAGTTTGCTCC
	PsCHK3-R1	AGTTCAGACGTGTGCTCTTCCGATCTATGGCAGAAGGATTCTTAGC

Table S3. Expression of *PsCHK* genes extracted from RNAseq Alves-Carvalho et al 2015. Colours indicate relative expression levels (red highest expression, yellow lowest expression).

	Stem	Peduncle	Leaf	Tendril	Shoot HN	Shoot LN	Apex	Flower	Pods	Seeds	Roots HN	Roots LN	Nodules 10dpi	Nodules mature
Psat7g004720 PsCHK1	16.081139	26.062143	5.423479	11.18758	6.29311	4.871085	9.260483	6.478823	7.579675	3.77462	8.16663	12.217054	13.38641	24.620002
Psat7g077280 PsCHK2	2.21267	5.066051	5.858519	3.396897	1.277805	1.095278	1.174534	2.872233	2.122538	2.644632	2.57008	3.735544	13.152336	6.41034
Psat5g097280 PsCHK3	14.078952	17.278118	17.11877	16.557223	10.850022	8.425631	8.508499	15.513222	4.580925	4.508327	12.242042	15.533663	6.612975	5.312018
Psat2g004360 PsCHK4	3.475653	5.713013	3.984072	4.625277	1.929093	1.822008	3.072027	2.480789	1.668	1.166047	2.605175	3.647236	0.738475	1.446594
